# NBS1 interacts with HP1 to ensure genome integrity

**DOI:** 10.1038/s41419-019-2185-x

**Published:** 2019-12-13

**Authors:** Giuseppe Bosso, Francesca Cipressa, Maria Lina Moroni, Rosa Pennisi, Jacopo Albanesi, Valentina Brandi, Simona Cugusi, Fioranna Renda, Laura Ciapponi, Fabio Polticelli, Antonio Antoccia, Alessandra di Masi, Giovanni Cenci

**Affiliations:** 1 Dipartimento di Biologia e Biotecnologie “C. Darwin”, Sapienza Università di Roma, Rome, Italy; 2 Fondazione Cenci Bolognetti/Istituto Pasteur Italia, Rome, Italy; 3 Dipartimento di Scienze, Università degli Studi Roma Tre, Rome, Italy; 4 Mechanisms of Transcription Laboratory, The Francis Crick Institute, 1 Midland Road, London, NW1 1AT UK; 5 Wadsworth Center, New York State Department of Health, Albany, NY 12201 US; 6 Istituto Nazionale di Fisica Nucleare, Sezione di Roma Tre, Rome, Italy; 7 Present Address: Telomeres and Telomerase Group, Molecular Oncology Program, Spanish National Cancer Research Center, Madrid, Spain

**Keywords:** Chromosomes, Diseases

## Abstract

Heterochromatin Protein 1 (HP1) and the Mre11-Rad50-Nbs1 (MRN) complex are conserved factors that play crucial role in genome stability and integrity. Despite their involvement in overlapping cellular functions, ranging from chromatin organization, telomere maintenance to DNA replication and repair, a tight functional relationship between HP1 and the MRN complex has never been elucidated. Here we show that the *Drosophila* HP1a protein binds to the MRN complex through its chromoshadow domain (CSD). In addition, loss of any of the MRN members reduces HP1a levels indicating that the MRN complex acts as regulator of HP1a stability. Moreover, overexpression of HP1a in *nbs* (but not in *rad50* or *mre11*) mutant cells drastically reduces DNA damage associated with the loss of Nbs suggesting that HP1a and Nbs work in concert to maintain chromosome integrity in flies. We have also found that human HP1α and NBS1 interact with each other and that, similarly to *Drosophila*, siRNA-mediated inhibition of NBS1 reduces HP1α levels in human cultured cells. Surprisingly, fibroblasts from Nijmegen Breakage Syndrome (NBS) patients, carrying the 657del5 hypomorphic mutation in NBS1 and expressing the p26 and p70 NBS1 fragments, accumulate HP1α indicating that, differently from NBS1 knockout cells, the presence of truncated NBS1 extends HP1α turnover and/or promotes its stability. Remarkably, an siRNA-mediated reduction of HP1α in NBS fibroblasts decreases the hypersensitivity to irradiation, a characteristic of the NBS syndrome. Overall, our data provide an unanticipated evidence of a close interaction between HP1 and NBS1 that is essential for genome stability and point up HP1α as a potential target to counteract chromosome instability in NBS patient cells.

## Introduction

The Heterochromatin Protein 1 (HP1) (also named HP1a in *Drosophila melanogaster* and HP1α in *Homo sapiens*) is a critical and conserved non-histone protein with essential roles in heterochromatin formation/maintenance and heterochromatin-related gene silencing^1^. Biochemical and structural studies revealed that ~200 amino acid HP1 protein consists of an *N*-terminal chromodomain (CD), separated from a related *C*-terminal chromoshadow domain (CSD) by a hinge region (H). The CSD mediates protein–protein interactions recognizing a pentapeptide motif, PxVxL^2,3^; this domain is also required for HP1 homodimerization and binding to a large set of proteins that maintain a high-order chromatin state. In contrast, the *N*-terminal CD binds dimethylated/trimethylated H3 tail on lysine 9, likely determining an epigenetic mark that compacts chromatin, restricts access of transcriptional factors, and results in transcriptional repression. The connecting H (hinge) region is responsible for binding both DNA and RNA. Its phosphorylation in *Drosophila* cells affects the HP1a domain’s function and localization^1,4–6^. Genome sequencing analysis has revealed that HP1 (as well as the other members of HP1 proteins family) is found in organisms from *Schizosaccharomyces pombe* to humans and appears to have similar functions in heterochromatin structure^7,8^.

The ability of HP1 to interact with several partners through the CSD renders HP1 a versatile chromatin protein involved in several functions. Mammalian HP1α accumulates at the site of DNA damage and loss of HP1α impairs the recruitment of RAD51, a key factor that promotes homologous recombination (HR) at double strand breaks (DSBs)^9^. Consistently, transient HP1 accumulation at DSBs has been proposed to ensure a proper HR^10–12^. Chromatin remodeling during HR repair is also regulated by Meiotic Recombination 11 (MRE11), RAD50, and Nijmegen Breakage Syndrome 1 (NBS1; also known as nibrin or NBN) (MRN) complex. This conserved complex allows the resection of damaged DNA and the docking of the complex with other DNA repair factors^11,12^.

Here we report an unanticipated and conserved functional relationship between HP1 and the MRN complex. We show that *Drosophila* HP1a binds the MRN complex and that its levels are reduced upon the loss of either Rad50, Mre11, or Nbs. However, HP1a-encoding *Su(var)2-5* gene genetically interacts only with *nbs* in maintaining chromosome integrity. Interestingly, also loss of human NBS1 reduces HP1α levels. Molecular docking simulations and experimental data indicate that the pentapeptide-like motif PGPSL found in NBS1 binds the CSD of HP1α similarly to other HP1α interactors. Unexpectedly, the expression of hypomorphic NBS1 protein variants in NBS patient cells causes the accumulation of HP1α and very likely delays its turnover. Interestingly, HP1α depletion in NBS cells decreases their hypersensitivity to ionizing radiation (IR). Overall, our data reveal that the NBS1–HP1 interaction preserves genome stability and that modulation of HP1α can affect NBS clinical features.

## Results

### HP1a physically interacts with the MRN complex

**Fig. 1:**
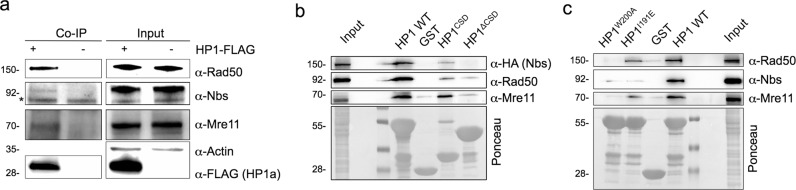
Drosophila HP1a physically interacts with the MRN complex. **a** Co-immunoprecipitation assay from HP1a-FLAG-overexpressing S2 cell extracts showing that HP1a precipitates endogenous Rad50, Mre11, and Nbs proteins (Input, 10% of total extract). The asterisk indicates aspecific bands. **b**, **c** Pulldown assays from Nbs-HA expressing Drosophila S2 cells with GST-tagged-full length HP1a (HP1a WT) and **b** GST-HP1a^CSD^ and GST-HP1a^∆CSD^ or **c** GST-HP1a^W200A^ and GST-HP1^I191E^ mutant proteins. Note that while Nbs has been revealed with a commercial anti-HA antibody, Rad50 and Mre11 have been detected with anti-Rad50- and anti-Mre11-specific antibodies generated in our laboratory. Ponceau staining shows the amount of each GST-tagged HP1a protein used in this assay. See text and “Materials and methods” for further details.

HP1 isoforms are known to interact with a large number of factors to fulfill their role in chromatin maintenance^1,6^. Proteomic approaches have also identified the MRN complex as a potential HP1 isoforms’ interacting factor^13,14^. However, a direct evidence of a functional relationship between HP1 proteins and the MRN complex has remained elusive. We sought to verify whether *Drosophila* HP1a could physically interact with Mre11, Rad50, or Nbs. By using extracts of HP1a-FLAG-expressing S2 cells, we found that HP1a is indeed able to precipitate the endogenous MRN complex, indicating for the first time that HP1a binds all components of the complex (Fig. 1a).

We expressed and purified from bacteria the recombinant full-length GST-HP1a (wild-type HP1a) and two HP1 truncations, namely, GST-HP1a^CSD^ containing only the CSD and GST- HP1a^∆CSD^ that lacks the C-terminal CSD domain. These proteins were used in standard GST-pulldown assays carried out from S2 cells extracts previously transfected with a Nbs-HA encoding vector. These experiments revealed that HP1a binds each component of the MRN complex through the CSD (Fig. 1b). We also tested whether mutations in the W200 and I191 residues of CSD, which are important for maintaining the canonical binding interface and promoting CSD dimerization^2^, respectively, could influence these interactions. We observed that, while both GST-HP1a^W200A^ and GST-HP1a^I191E^ mutant proteins failed to efficiently pull down Nbs (detected by the anti-HA antibody), the binding of endogenous Mre11 and Rad50 was influenced by the W200A mutation but only marginally by the I191E (Fig. 1c). This indicates that HP1a requires both CDS dimerization and a canonical binding interface to interact with Nbs, while its binding to Mre11 and Rad50 relies mainly on an intact C-terminal portion.

### Loss of MRN reduces HP1a levels

**Fig. 2:**
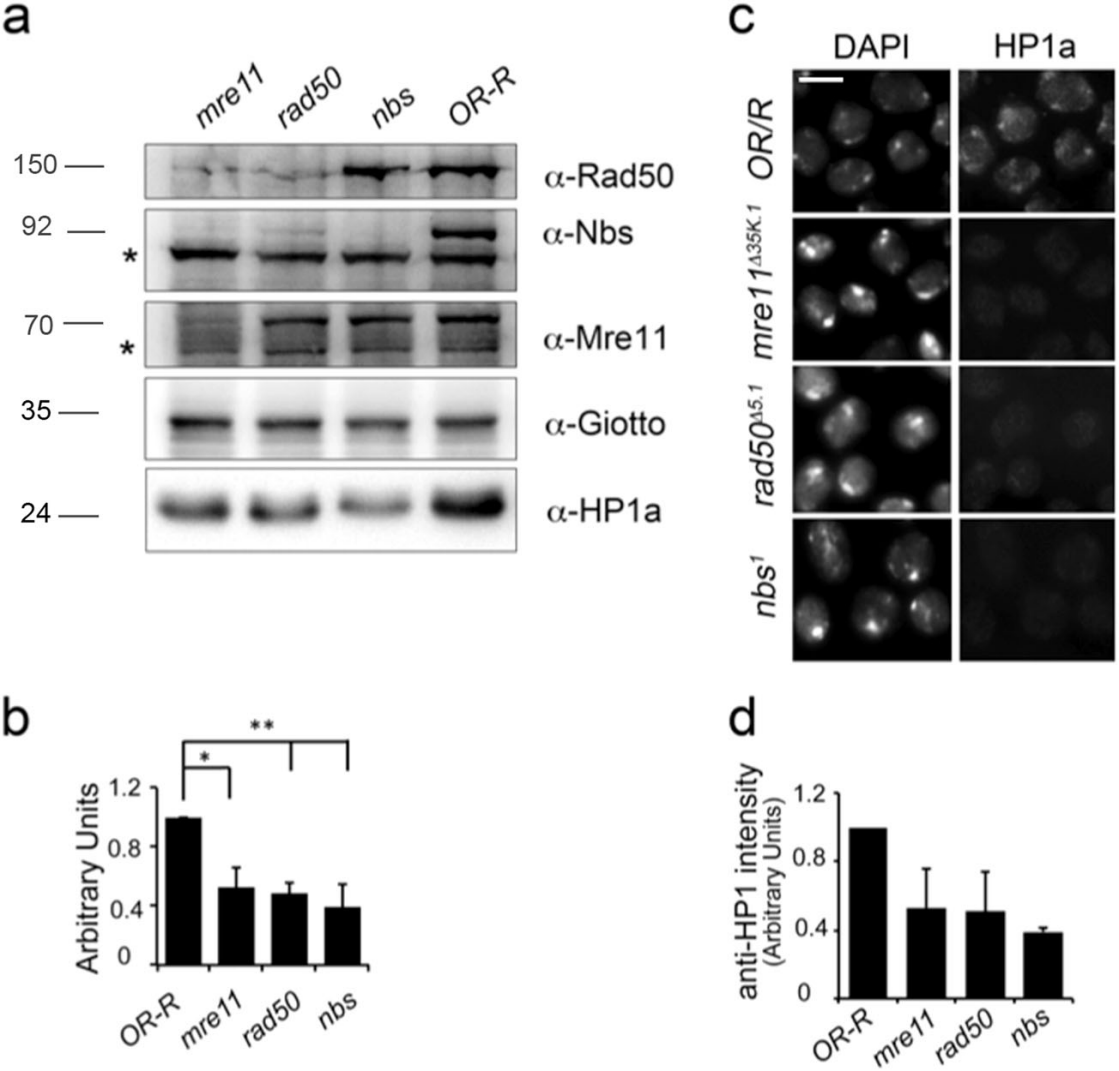
Loss of the Drosophila MRN complex affects HP1a levels. **a** WB analysis from Oregon R wild-type and *nbs*^*1*^, *rad50*^*∆5.1*^, and *mre11*^*Δ35K.1*^ mutant larval brains showing that HP1a levels are reduced when any of the MRN complex member is depleted. The anti-Giotto antibody has been used as a loading control^69^. The asterisks (*) indicate aspecific bands. Note the absence of Nbs, Rad50, and Mre11 bands in the corresponding *nbs*^*1*^, *rad50*^*∆5*.*1*^, and *mre11*^*Δ35K.1*^ loss-of-function mutants that also reveals the specificity of the our anti-Nbs, anti-Mre11 (this study), and anti-Rad50 antibodies^15^. **b** Quantification analysis of HP1a reduction from at least four independent WBs. (*t* Student’s Test; *p < 0.05; **p < 0.01). **c** Localization of HP1a in *nbs*^*1*^, *rad50*^*∆5*.*1*^, and *mre1*^*1Δ35K.1*^ mutant brain metaphases. Bar: 10 μm **d** Quantification of anti-HP1a immunolocalization (*t* Student’s Test; *p < 0.01. Bars indicate ±S.D.

We wanted to verify whether HP1a levels were affected in the severely hypomorphic *nbs*^*1*^, *rad50*^*∆5.1*^, or *mre11*^*Δ35K.1*^ third instar lethal mutants. Western blot (WB) analysis on mutant larval brains revealed that HP1a levels were drastically reduced (>50%) in all *MRN* mutants (Fig. 2a, b). Consistently with our WB, HP1a immunofluorescence (IF) in *nbs*^*1*^, *rad50*^*∆5.1*^, and *mre11*^*Δ35K.1*^ mutant chromosomes was also significantly reduced (~50%) with respect to controls (Fig. 2c) indicating that a decrease of HP1a levels resulted also in a reduced HP1a localization. Moreover, quantitative reverse transcription polymerase chain reaction (qRT-PCR) on RNA extracted from *nbs*^*1*^, *rad50*^*∆5.1*^, and *mre11*^*Δ35K.1*^ homozygous larval brains revealed that *Su(var)2-5 (HP1a)* mRNA levels were only partially affected by depletion of each MRN complex member (Supplementary Fig. 4) suggesting that MRN generally regulates HP1a protein stability rather than its transcription.

**Fig. 3:**
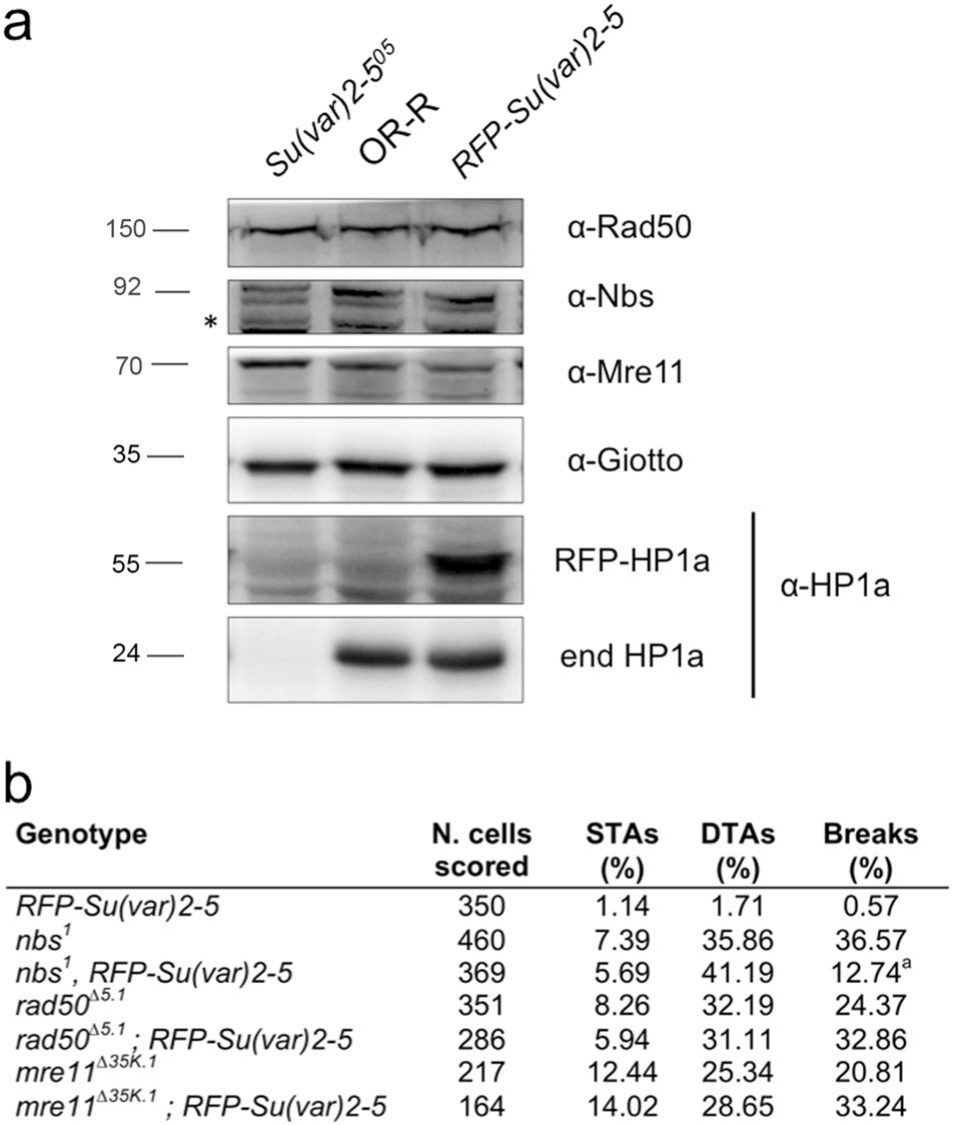
HP1a does not affect the expression of the MRN complex. **a** WB from *Su(var)2-5*/*HP1a* mutant extracts and from control (OR) or HP1a-expressing *Su(var)2-5-RFP* protein extracts, revealed with anti- MRN complex and anti-HP1 antibodies. Anti-actin and anti-Giotto have been used as loading controls. The asterisks (*) indicate aspecific bands. **b** Quantification of chromosome break frequency in *MRN* mutants expressing HP1a-RFP (^a^*t* Student’s Test; *p* < 0.01). STAs = Single Telomere Attachments; DTAs = Double Telomere Attachments.

WB from *Su(var)2-5* mutant combinations as well as from *Su(var)2-5* interfered larvae showed no variations in the Nbs, Rad50, or Mre11 protein levels (Fig. 3 and Supplementary Fig. 1) thus excluding a mutually dependent relationship between HP1a and the complex. Collectively, these results show that MRN might act as a positive regulator of HP1a levels. It is worth noting that HP1a levels in *nbs*^*1*^ mutants were partially restored to a normal steady-state level upon treatment with the lysosomal inhibitor chloroquine but not with the proteasome inhibitor MG132 (Supplementary Fig. 2). This suggests that, at least in *Drosophila*, the Nbs–HP1a interaction prevents HP1a lysosomal-dependent degradation.

### Restoring HP1a normal levels reduces chromosome breakage induced by depletion of Nbs but not of Rad50 and Mre11

To understand whether HP1a could modulate the role of the MRN complex in the maintenance of chromosome integrity in flies^15–18^ (Supplementary Fig. 3), we restored HP1a normal levels expressing HP1a-RFP in *nbs*^*1*^, *rad50*^*∆5.1*^, and *mre11*^*Δ35K.1*^ mutants. Whereas the expression of HP1a had no effect on the chromosome break frequency of *rad50*^*∆5.1*^ and *mre11*^*Δ35K.1*^ mutant cells, it strongly lowered (~5-fold) chromosome breakage of *nbs*^*1*^ mutants (Fig. 3b). Although HP1a is physically associated with the MRN complex, *Su(var)2-5* genetically interacts only with *nbs* suggesting a close functional relationship between these two factors. Surprisingly, HP1a-RFP expression did not influence the frequency of *nbs*^*1*^, *rad50*^*∆5.1*^, and *mre11*^*Δ35K.1*^ telomeric fusions indicating that uncapped telomeres generated by the loss of MRN are not simply caused by a reduction of HP1a at chromosome ends.

### Functional relationship between human NBS1 and human HP1

**Fig. 4:**
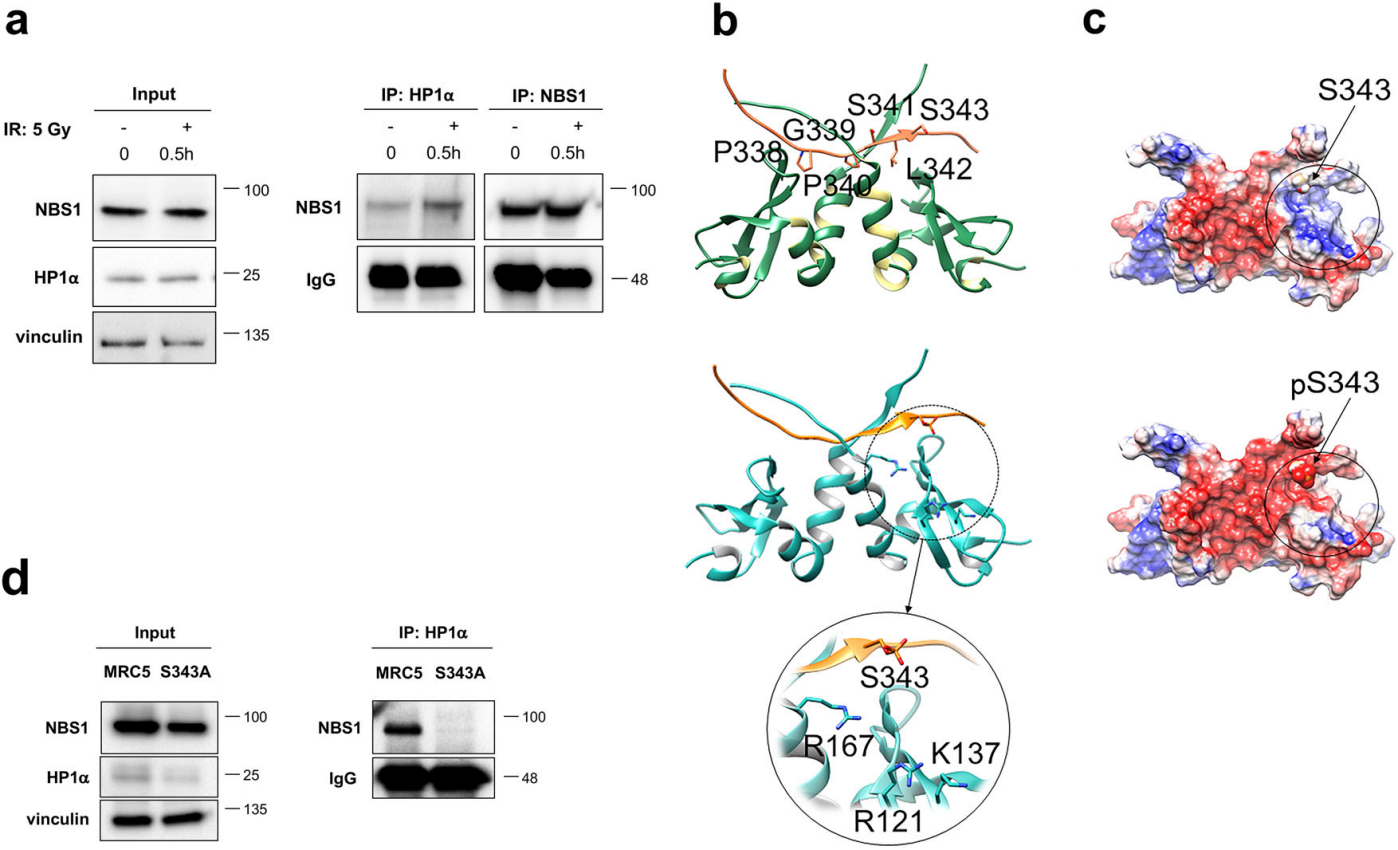
NBS1 interacts with HP1α following DNA damage. **a** WB analysis of cellular extracts from untreated and irradiated MRC5 fibroblasts after immunoprecipitation (IP) with either anti-HP1*α* or anti-NBS1 antibodies. MRC5 cells were irradiated with 5 Gy of X-rays and harvested after 0.5 h. One milligram of total protein extracts were immunoprecipitated and 10 μg of total protein lysate (1%) were loaded as inputs. Membranes were probed with anti-HP1α or anti-NBS1 antibodies. Note that HP1α cannot be revealed in the IP of NBS1 since its molecular weight of *~*22 kDa is the same as the light chain of immunoglobulins. Total IgG levels and vinculin were used as loading control for immunoprecipitates and input, respectively. **b** Top: Structural complex between HP1α (sea green) and the NBS1 peptide containing the PGPSL sequence (coral) that allows the recognition of HP1α. Down: Structural complex between HP1α (sky blue) and the NBS1 peptide containing the PGPSL sequence (orange) in which the S343 residue is phosphorylated. **c** Electrostatic potential surface representation of the structural complex HP1α–NBS1 peptide in which the S343 residue is either unphosphorylated (top) or phosphorylated (down) (red, −2 kT/e; white, 0.0 kT/e; blue, +2 kT/e). **d** WB from control MRC5 cells and NBS1 cells carrying the S343A point mutation, immunoprecipitated with anti-HP1α antibody. Ten micrograms of total protein lysate (1%) were loaded as input. Membranes were blotted with anti-NBS1; total IgG levels and vinculin were used as loading controls for immunoprecipitates and input, respectively.

We asked whether a NBS1 and HP1 functional relationship also occurs in human cells. NBS1 consists of three regions: the *N-*terminal region containing the forkhead-associated (FHA) domain and two breast cancer 1 (BRCA1) carboxy-terminal (BRCT) tandem domains^19–22^; the central region containing a consensus sequence encompassing the Ser343 residue that, together with the Ser278 residue located within the BRCT2 domain, is phosphorylated by ATM kinase in response to IR^23–26^; the *C*-terminal region containing the MRE11- and ATM-binding motifs^19,27^. We found that Sepharose-bound anti-NBS1 antibody is able to pulldown endogenous HP1α in human MRC5 fibroblasts confirming that the NBS1–HP1α physical interaction is evolutionarily conserved (Fig. 4a). Although the total levels of NBS1 and HP1α were not affected by DNA damage, a twofold increase of NBS1–HP1α complex levels were observed in irradiated MRC5 cells (Fig. 4a). This suggests that an increment of NBS1–HP1α interaction is likely to occur as a part of the DNA damage response (DDR) following irradiation.

The analysis of NBS1 amino acid sequence revealed that downstream the tandem BRCT domains lies a PGPSL stretch (amino acids 338–342) that is reminiscent of the HP1 PxVxL motif required for the binding of HP1 to several partners^2,3^. Molecular docking simulations performed between HP1 CSD and the NBS1 peptide containing the PGPSL sequence predicted that the peptide binds in a crevice, formed by two β-strands belonging to each chain of the HP1 protein, in which other HP1 interactors are known to bind^28^ (Fig. 4b). Docking simulations of the NBS1 peptide phosphorylated on S343 onto HP1α predicted an identical binding mode (Fig. 4c). Interestingly, in the simulated complex the negative charge of phosphorylated S343 is hosted in a positively charged pocket, formed by the network of charged residues R121, K137, and R167 (Fig. 4b, c). The electrostatic potential in this region is highly positive in the presence of the non-phosphorylated peptide while it becomes more neutral if the peptide is phosphorylated (Fig. 4c). Thus phosphorylation of NBS1 S343 is predicted to promote NBS1–HP1α binding through favorable electrostatic interactions. In agreement with this, co-immunoprecipitation (co-IP) from NBS cells (that do not express the wild-type NBS1, see below) and transfected with a NBS1^S343A^-expressing construct^29^ showed that NBS1^S343A^ fails to interact with HP1α (Fig. 4d). This indicates that the 338–342 stretch of NBS1 is the likely HP1α-binding candidate region. Of note, the NBS1^S343A^-expressing NBS cells elicited also lower HP1α levels compared to wild-type MRC5 cells (Fig. 4d). In addition, the alkaline phosphatase (AP) treatment abolished the NBS1–HP1α interaction further suggesting that phosphorylation may act as a key post-translational modification that modulates this interaction (Supplementary Fig. 5).

### HP1α levels are NBS1 dependent

**Fig. 5:**
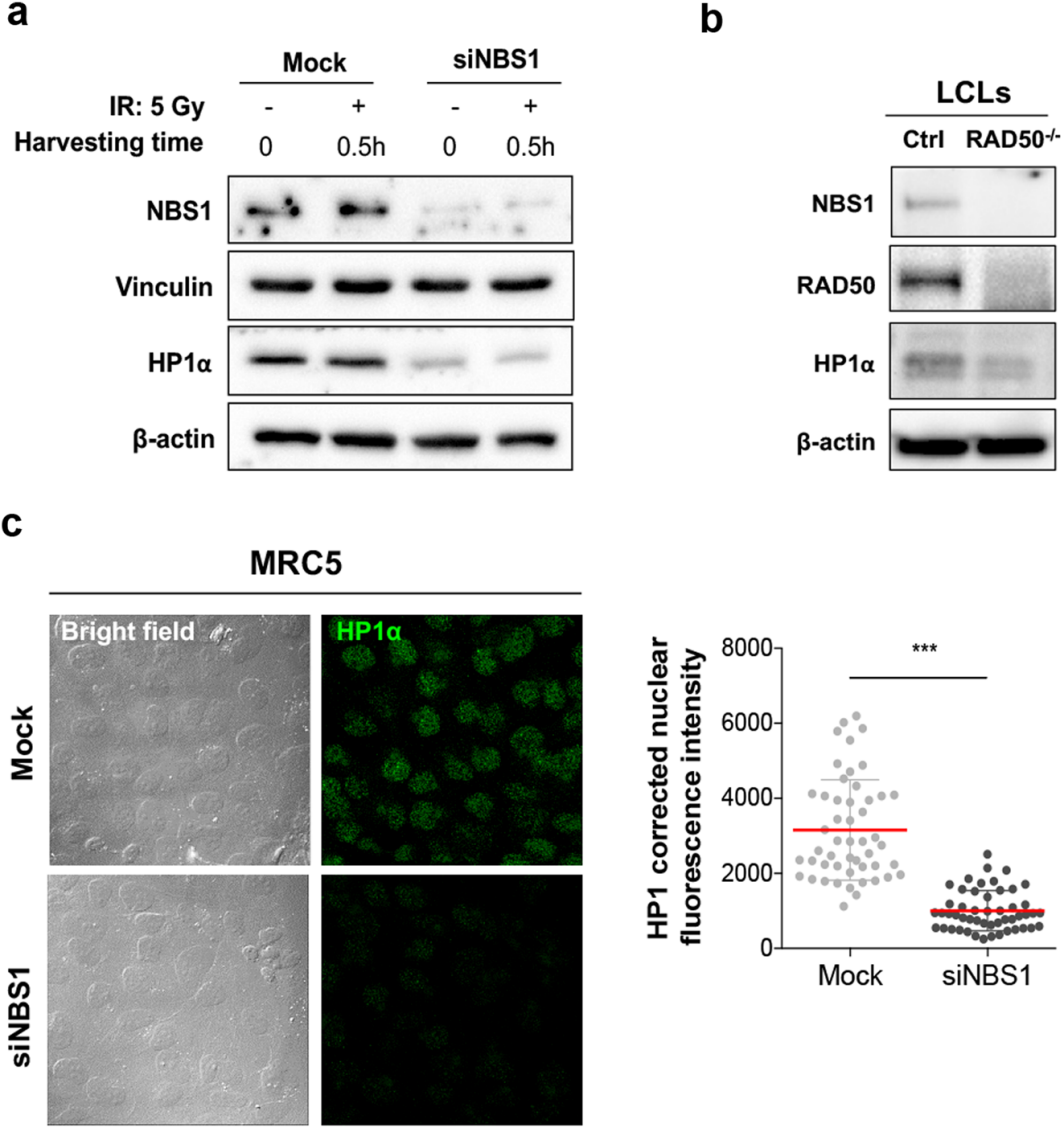
NBS1 regulates the stability of HP1α. **a** WB from both untreated and irradiated mocked and siNBS1– MRC5 cell extracts. Cells were irradiated with 5 Gy of X-rays and harvested after 0.5 h. Ten micrograms of total protein lysates were analyzed by WB. Membranes were probed with anti-HP1α and anti-NBS1 antibodies; vinculin and β-actin were used as loading controls. **b** WB from extracts of lymphoblastoid cells (LCLs) established from an healthy donor (Ctrl) and from a patient carrying two germline mutations in the *RAD50* gene (RAD50^−/−^)^30^. Membranes were probed with anti-HP1α, anti-NBS1, and anti-RAD50 antibodies; β-actin was used as loading control. **c** Left: representative images of the immunofluorescence analysis of HP1α (Alexa Fluor 480, green fluorophore green) protein levels in mocked and NBS1-silecenced MRC5 cells. Bar: 20 μm. Right: Distribution of HP1α corrected nuclear fluorescence intensity in mocked and NBS1-silecenced MRC5 cells. The horizontal lanes indicate the mean values derived from the analysis of 100 cells/experimental point in three independent experiments ±S.D. (*t* Student’s Test; ***p < 0.001).

WB analysis revealed that depletion of NBS1 caused a 3.3-fold reduction of HP1α levels in NBS1-siRNA MRC5 cells compared to mock-treated cells (Fig. 5a), suggesting that, similarly to flies, NBS1 regulates HP1α levels also in human cells. The same reduction was also observed upon X-ray treatment confirming that NBS1-mediated regulation of HP1α is independent of DDR (Fig. 5a). Noteworthy, the RAD50-mutated lymphoblastoid cells, which exhibit low levels of NBS1 compared to controls^30^, also showed reduced levels of HP1α protein compared to controls (Fig. 5b). This regulation was also confirmed by HP1α immunostaining on NBS1-depleted fibroblasts, which revealed a threefold reduction of HP1α staining intensity compared to mock cells (Fig. 5c). Moreover, qRT-PCR on mRNA isolated from the same siNBS1-interfered fibroblasts indicated that *HP1α* mRNA levels were not reduced upon depletion of NBS1 but rather slightly increased with respect to mock-treated cells (Supplementary Fig. 6a). To verify whether depletion of NBS1 affected HP1α turnover, we performed a cycloheximide (CHX) chase assay in both mock- and siNBS-treated MRC5 cells. Interestingly, we found that HP1α levels of siNBS1-interfered cells after 1 h of CHX increased with respect to untreated cells (0 h). Nonetheless, after 2 and 4 h CHX treatment in both mock- and siNBS1-treated HP1α levels slightly diminished and significantly decreased after 8 h CHX treatment (Supplementary Fig. 7). Although the reason why HP1α increased at 1 h CHX treatment in NBS1-depleted cells is not quite clear, we can speculate that it could result from CHX-dependent lysosome inhibition^31–33^ that could potentially rescue HP1α degradation occurring very likely through lysosomal proteolysis. This finding is also supported by our observation that low HP1a levels in Drosophila *nbs* mutants are restored to normal upon treatment with the lysosome inhibitor chloroquine (Supplementary Fig. 2) and confirms our hypothesis that NBS–HP1α interaction could prevent lysosome-dependent degradation of HP1. Collectively, these findings suggest that the NBS1 role as a positive regulator of HP1α protein levels represents an evolutionary conserved function.

### HP1α is misregulated in NBS patient-derived fibroblasts

**Fig. 6:**
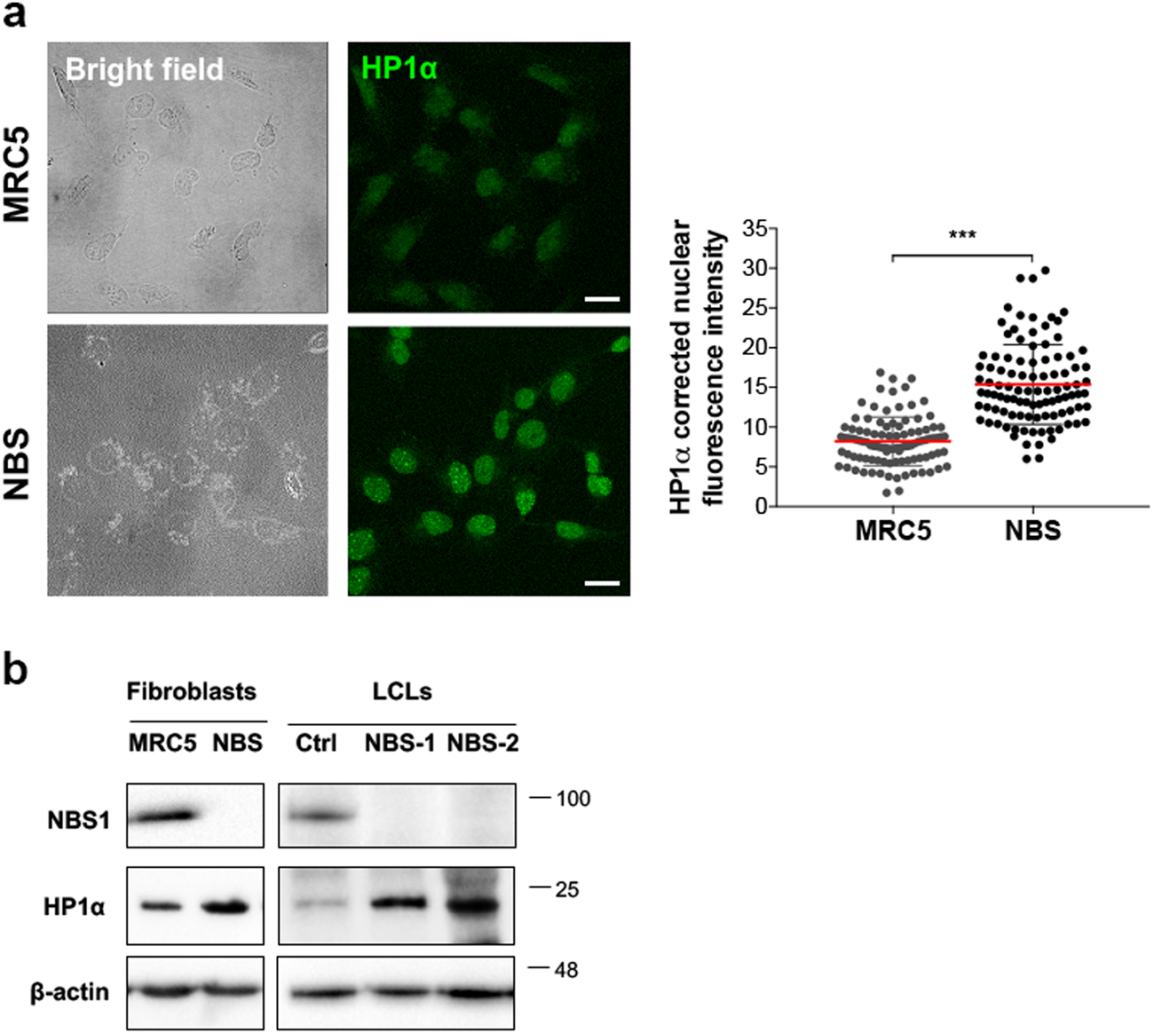
HP1α accumulates in NBS cells. **a** Left: representative images of the immunofluorescence analysis of HP1 levels (green) in MRC5 and NBS cells. Bar: 20 μm. Right: Distribution of HP1α corrected nuclear fluorescence intensity in MRC5 and NBS1 cells. The horizontal lanes indicate the mean values derived from the analysis of 100 cells/experimental point in three independent experiments ±S.D. (*t* Student’s Test; ***p < 0.001). **b** WB analysis of HP1α levels in fibroblast and lymphoblastoid cells (LCLs). The levels of HP1α and NBS1 in NBS cells were compared to their corresponding controls (i.e., MRC5 for NBS1 fibroblasts; Ctrl for NBS1–1 and NBS1–2 lymphoblastoid cells); β-actin was used as the loading control.

We next asked whether HP1α levels were also affected in human fibroblasts derived from NBS patients who carried the NBS1 657del5 mutation (NBS1; OMIM #2512609)^34,35^. This mutation, identified in 90% of NBS patients, generates a predicted 26-kDa N-terminal fragment (hereafter p26-NBS1) and a 70-kDa C-terminal fragment (hereafter p70-NBS1). The 26‐kDa fragment includes the FHA and the BRCT1 domains (amino acids 1–218), whereas the 70‐kDa fragment contains the region encompassing the BRCT2 domain and the C‐terminal region (amino acids 221–754). The p70-NBS1 is produced by internal translation initiation within the *NBS1* mRNA using an open reading frame generated by the 657del5 frameshift. Both p26- and p70-NBS1 fragments possess residual function^36,37^. This mutation leads to the onset of the NBS genetic disease that shares a number of features with ataxia-telangiectasia (A-T), such as a high sensitivity to IR and predisposition to cancer^27^. Unexpectedly, IF on fixed NBS1 cells revealed a two-fold increase of HP1α intensity compared to MRC5 control cells (Fig. 6a). Consistently, WB analysis from NBS fibroblasts and lymphoblastoids showed an approximate three-fold to five-fold increase of HP1α levels compared to corresponding controls (Fig. 6b). However, no differences in levels of HP1α mRNAs were observed between MRC5 and NBS cells (Supplementary Fig. 6b) indicating that high HP1α levels are not due to increased transcription. This suggests that, while loss of NBS1 reduces HP1α levels, the presence of dysfunctional NBS1 fragments, as in NBS patients, could enhance HP1α protein stability.

**Fig. 7:**
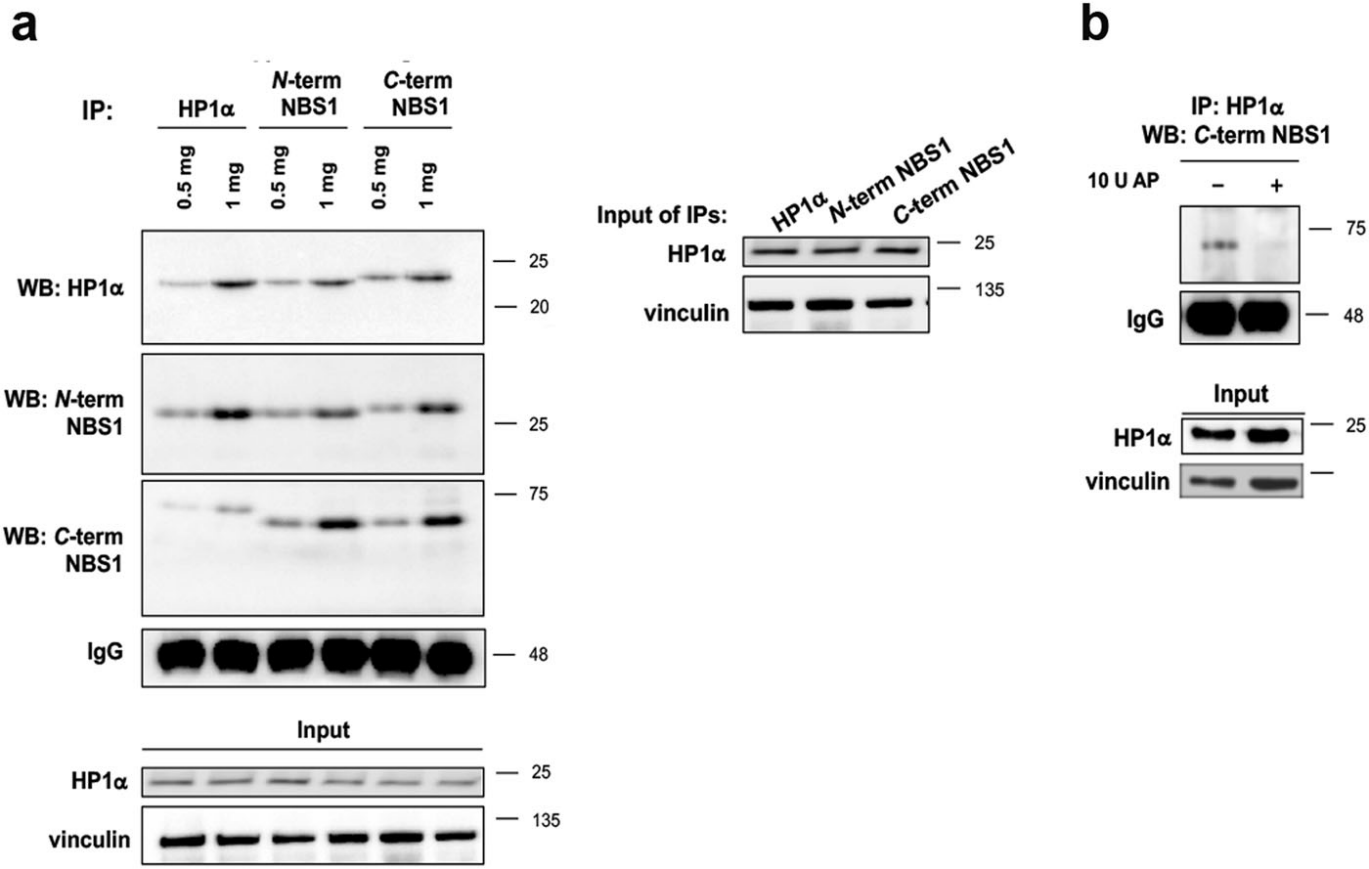
NBS1 fragments arising from the 657del founder mutation in NBS1 patients increase HP1α levels. **a** WB analysis from total protein lysate derived from NBS cells, immunoprecipitated with either anti-HP1α or with two different anti-NBS1 antibodies directed against the *N*- and the *C*-terminus of NBS1. Membranes were probed with the anti-HP1α or the two anti-NBS1 antibodies. The corresponding inputs (1%), probed with anti-HP1α and anti-vinculin antibodies, are shown below. **b** One milligram of whole-protein lysate obtained from NBS1 cells were incubated in the absence (−) or presence (+) of 10 U alkaline phosphatase (AP). The untreated and dephosphorylated samples were immunoprecipitated with anti-HP1α antibody and membranes were probed with anti-NBS1 antibody directed against the *C*-terminus of NBS1. Total IgG levels and vinculin were used as loading controls for immunoprecipitates and input, respectively.

Co-IP assays from NBS fibroblasts with anti-*N*-terminal and anti-*C*-terminal NBS1 antibodies as well as with anti HP1α showed that both p26-NBS1 and p70-NBS1 maintained the ability to interact with HP1α (Fig. 7). However, while p26-NBS1 pulled down HP1α with the expected size, the HP1α associated with p70-NBS1 showed a molecular weight slightly higher than expected. We can speculate that these different migration patterns likely depend on potential post-translational modifications that both proteins undergo in order to interact with each other. Furthermore, AP treatment completely abolished the p70-NBS1:HP1α interaction suggesting that phosphorylation of HP1α, p70-NBS1, or both is indeed required for these interactions (Fig. 7b).

### Reduction of HP1α in human NBS1 fibroblasts decreases the hypersensitivity to IR

**Fig. 8:**
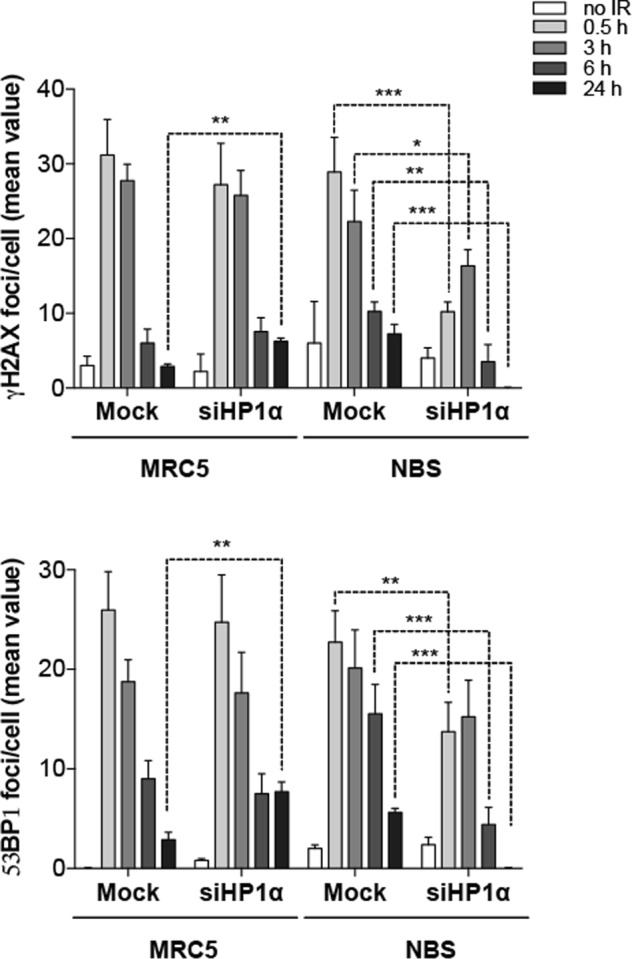
HP1α inhibition reduces IR-induced DSBs in NBS cells. Analysis of DSB repair kinetics of γH2AX and 53BP1 in mock- and HP1α-silenced MRC5 and NBS cells, irradiated with 1 Gy of X-rays and harvested after 0.5, 3, 6, and 24 h. Graphs express the mean number of either γH2AX or 53BP1 foci/cell derived from the analysis of 100 cells/experimental point, in three independent experiments ±S.D. (*t* Student’s Test; *p < 0.05, **p < 0.01, ***p < 0.001).

To verify whether the increased HP1α levels in NBS cells could in part explain the cell radiosensitivity associated with the syndrome, we asked whether depletion of HP1α could counteract the NBS cell response to IR. We thus evaluated the kinetics of disappearance of γH2AX and 53BP1 foci in mock- or HP1α-interfered MRC5 and NBS fibroblasts, following exposure to 1 Gy of X-rays. We found that, in MRC5 cells fixed at different post-irradiation times, HP1α silencing resulted in a persistence of a higher number of γH2AX and 53BP1 foci per cell at 24 h from IR compared to mocked-treated MRC5 cells. On the contrary, HP1α silencing in NBS cells resulted in a statistically significant reduction (>50% at 4 h post-irradiation time) of number of γH2AX and 53BP1 foci per cell at all the time points analyzed with respect to mock-transfected cells (Fig. 8 and Supplementary Fig. 8). This finding suggests that HP1α accumulation in NBS cells can indeed account for their X-ray hypersensitivity phenotype and opens the possibility in the future to consider HP1α a potential target to counteract the NBS-associated chromosome instability.

## Discussion

Our data provide a compelling evidence of an unanticipated and conserved interaction between NBS1 and HP1 in both Drosophila and human cells. We showed that Drosophila HP1a physically interacts with the MRN components and that the whole MRN complex acts as a positive regulator of HP1a. However, two lines of evidence suggest that Drosophila HP1a regulation by Mre11 and Rad50 occurs differently from that mediated by Nbs. First, the binding of HP1a to Nbs requires both the intact canonical binding interface and the CSD dimerization, while that to Rad50 and Mre11 relies mainly on an intact C-terminal portion. Second, the restoring of normal levels of HP1a drastically reduces the number of spontaneous chromosome breaks observed in *nbs*, but not *rad50* or *mre11*, mutants. Thus, based on both genetic and physical interactions, we argue that Nbs plays a primary role in the functional relationship between Drosophila MRN complex and HP1a. We also showed for the first time that Drosophila Nbs levels are reduced in *rad50* and *mre11* mutants (Fig. 3), similarly to cells from ATLD and Rad50-mutated patients^30,38^. Thus it is conceivable that the HP1a reduction in either *rad50* or *mre11* mutant cells is more an indirect effect of the Nbs impairment rather than a direct consequence of a perturbed interaction between Rad50, Mre11, and HP1a. Finally, the reduction of human HP1α levels in NBS1-depleted cells suggests that NBS1-mediated regulation of HP1 is evolutionarily conserved.

The reason why the members of the Drosophila MRN complex behave differently in the regulation of HP1a is not clear. However, this is not unexpected as Nbs and the Mre11/Rad50 complex were shown to play partially independent roles in Drosophila telomere protection^16^. In addition, human NBS1 and the MRE11/RAD50 components play separate roles in both ATM activation and ATM-mediated phosphorylation^39,40^ and exhibit distinct localization at telomeres^41^.

Like Rad50, we showed that Drosophila Nbs localizes diffusely on mitotic chromosomes (Supplementary Fig. 10), reinforcing the view that Drosophila Nbs and most probably the entire MRN complex can be considered as a prominent chromatin factor, which stably resides on chromatin where it co-localizes with HP1a. Moreover, we have shown that HP1a physically interacts with Nbs and that this interaction requires the dimerization of HP1a and the integrity of its binding interface, as expected for most HP1-binding partners.

Our molecular docking simulations between HP1 CSD and the NBS1 PGPSL sequence predicted that the PxVxL-like pentapeptide binds in a crevice formed by two β-strands belonging to each chain of the HP1 protein. We argue that this binding reflects a physiological interaction. Indeed, intrinsically disordered (ID) regions are present in at least 30% of the eukaryotic proteome and are enriched in chromatin-associated and/or DNA repair proteins^42–45^. IDs endow proteins with dynamic flexibility, intermolecular recognition properties, and the ability to integrate signals from various intracellular pathways^46–51^. These features are indeed fundamental for a protein in order to bind different bulky adducts and also interact with other proteins^47,52^. Moreover, ID regions undergo post-translational modifications that serve as decision points in signaling pathways^53^. We also predicted that S343 phosphorylation, within the NBS1 pentapeptide, favors the formation of the NBS1-HP1α complex and consistently that a unphosphorylable S343 impairs the NBS1–HP1α interaction. We can thus conclude that the 338–342 stretch of NBS1 ID region is the favorite HP1α-interacting region.

How NBS1 secures HP1 levels is unclear. Our quantitative real-time PCR (qPCR) analyses ruled out the possibility that NBS1 could be mainly involved in the regulation of HP1 mRNAs. Chloroquine treatment in Drosophila revealed that Nbs could in part prevent HP1a premature lysosome-dependent degradation by autophagy. Our CHX experiments likely support this finding. Degradation of HP1α through the autophagy pathway has been described to occur in human cells to guarantee efficient completion of DDR repairs^54^. It is therefore possible that the binding of HP1 with NBS1 could prevent this interaction to avoid unwanted degradation of HP1.

The finding that additional copies of HP1a reduce spontaneous chromosome breaks in *nbs* mutants indicates that the DNA repair activity of Nbs relies on a proper function and/or localization of HP1a on chromatin. We have also observed that the levels of HP1α–NBS1 complex increased following IR of human cells. It is known that upon DSB induction, NBS1 forms a macromolecular complex with a number of proteins in order to promote DSB resection during HR^55^. Thus it is possible that the interaction of HP1α with NBS1, together with the reported interaction of HP1α with BARD1, FANCJ, and CtIP^56–58^, is required to fulfill the DDR.

The HP1α accumulation in NBS cells came as a surprise, which apparently contrasts the observations of NBS1-depleted cells. We demonstrated that the p26- and p70-NBS1 fragments, resulting from the 657del5 mutation, still exhibit the capability to bind HP1α. This is in line with previous proteomic observations indicating that p26- and p70-NBS1 still maintain the capability to interact with several chromatin factors^59^. We believe that p70-NBS1 binds HP1α through the HP1α-binding pentapeptide retained in the fragment while the p26-NBS1 fragment interacts with HP1α as a consequence of the dimerization of its BRCT1 domain with the p70-NBS1 BRCT2 domain. However, it cannot be excluded that the tandem BRCT domains of NBS1 play per se a role in the interaction with HP1α. NBS1 protein carrying the R215W missense mutation, which perturbs the relative orientation of the tandem BRCT domains^22,60^, fails to interact with HP1α. These findings suggest that the tandem BRCT domains could contribute, along with the pentapeptide, to the interaction with HP1α. This is reminiscent of what observed in BARD1 in which the conserved HP1α-binding pentapeptide is located within the BRCT domain and allows the interaction with the CSD of HP1α^56,57^. Differently from NBS1 silencing, the interaction of p26- and p70-NBS1 fragments with HP1α results in an accumulation of HP1α through mechanisms that remain unclear. Increased HP1α levels are not due to an elevated transcription of *HP1α* gene. A plausible explanation is that HP1 is translated more abundantly in these cells. Further studies are needed to clarify this point.

Finally, we reported that small interfering RNA (siRNA)-mediated depletion of HP1α in NBS cells alleviates the NBS hypersensitivity to X-ray-induced DSBs. This is an unanticipated finding that highlights a clear cause–effect relationship between HP1α and NBS cell instability. HP1α accumulation has been proven to cause deleterious effects on cell survival in different systems and shows a significant correlation with tumor progression^61,62^. Therefore, it is not unexpected that part of the genome instability observed in NBS cells could arise as a consequence of high HP1α levels. An accumulation of HP1α on chromatin could indeed perturb chromatin compaction contributing to epigenetic-mediated alteration of DDR gene expression and/or hampering a proper recruitment of DDR factors upon irradiation. Whatever the mechanisms, our observations might provide some clues on a potential HP1α-based route to counteract some of the NBS chromosome defects. Overall, our data reveal that NBS1 interacts with and regulates HP1 and that their functional relationship is crucial for the NBS1 activity in preserving genome integrity.

## Materials and methods

### Drosophila strains, crosses, and germline transformation

The *nbs*^*1*^, *nbs*^*2*^, and *rad50*^*∆5.1*^ *mre11* ^*Δ35K.1*^ mutations and the *Su(var)205* mutant alleles were previously described^15,16,63,64^. The UAS *Su(var)2-5RNAi* (v104893) and *Act5-Gal4* (*P{Act5C-GAL4}25FO1/CyO*) expressing lines were obtained from Vienna *Drosophila* RNAi Center and Bloomington Stock Center, respectively. The *Su(var)2-5-RFP* is a transgenic line that contains the HP1a-RFP-expressing construct on chromosome 3^65^. *Oregon R* (*OR)* has been used as a wild-type control. The *UASnbsGFP* line has been generated by standard germ-line transformation. This strain carries the *nbs* cDNA-containing transgene on chromosome 2 that was generated by the Gateway strategy (Invitrogen, Carlsbad, CA, USA) using a pPWG as destination vector (DGCR, Indiana University). The *nbs* CDS was amplified from an imaginal disc cDNA library using primers *nbs_FW* and *nbs_RV* (Supplementary Table 1). The *actGal4 UAS-GFP-nbs* line was generated by recombination between two chromosome 2 carrying *actGal4* and *UASnbsGFP*. Single recombinants were selected owing to the green fluorescent protein (GFP) expression in larvae and kept as an *actGal4 UAS-GFP-nbs/CyO* stock. *nbs*^*1*^*,* *Su(var)205-RFP/TM6C* line was generated by crossing *w; nbs*^*1*^*/TM6C* with homozygous *Su(var)25-RFP* flies; heterozygous F1 *w; nbs*^*1*^*/Su(var)25-RFP* females were crossed to *w; Ap*^*Xa*^*/TM6C* and recombinant third chromosomes were isolated by crossing single F2 males with *w*; *Ap*^*Xa*^*/TM6C* females and balanced over *TM6C* chromosome. The *rad50*^*∆5*.1^/*CyOGFP; Su(var)25-RFP* and *mre11* ^*Δ35K.1*^*/CyOGFP*; *Su(var)2-5-RFP* lines were obtained by crossing *rad50*^*∆5*.1^/*CyOGFP*; *MKRS/TM6B* and *mre11* ^*Δ35K.1*^/*CyOGFP; MKRS/TM6B* with *w; Sco/CyOGFP; Su(var)2-5-RFP* flies. All balancer chromosomes are described in Flybase.

### Drosophila cell culture and transfection

*Drosophila* S2 cells were cultured at 25 °C in Schneider’s insect medium (Sigma-Aldrich, Saint Louis, MO, USA) supplemented with 10% heat-inactivated fetal bovine serum (FBS) (Gibco, Waltham, MA, USA). For transfection, S2 cells were plated at the concentration of 3 × 10^6^ cells/mL in a six-well culture dish. After 24 h, cells were transfected with Nbs-HA or HP1-FLAG encoding plasmids (pAWHNbs and pAWFHp1, respectively) by using Effectene transfection reagent (Qiagen, Venlo, The Netherlands) according to the manufacturer’s instructions. After 72 h from transfection, cell cultures were harvested, washed two times with phosphate-buffered saline (PBS), and pellets were processed for protein extract preparation.

### Human cell lines, culture methods, and siRNA transfection

The SV40-transformed fibroblasts were established from a normal donor (here named MRC5), from the GM7166VA7 patient affected by NBS homozygous for the 657del5 mutation in the *NBS1* gene (here named NBS; ref. ^35^), and from the NBS cell line stably expressing the S343A point mutation (kindly provided by Dr. K. Komatsu, Kyoto University, Kyoto, Japan). Cells were grown in Dulbecco Modified Eagle’s medium (Biowest, Nuaillé, France) supplemented with 10% FBS (Corning, Corning, NY, USA), 100 μg/mL penicillin and streptomycin (Sigma-Aldrich), and 2 mM L-glutamine (Biowest). Lymphoblastoid cells were derived from one normal individual (Ctrl), two NBS patients (NBS-1 and NBS-2), and one patient carrying two germline mutations in the *RAD50* gene^30^. Lymphoblastoids were cultured in RPMI-1640 (Biowest) supplemented with 10% heat-inactivated fetal calf serum (Gibco), 100 μg/mL penicillin and streptomycin (Sigma-Aldrich), 2 mM l-glutamine (Biowest), and 0.5% non-essential amino acids. All the cells were grown at 37 °C in a 5% CO_2_ atmosphere.

The transient silencing of NBS1 or HP1α in MRC5-SV40 cells was performed by the double transfection method using 5 nM of the Mission® esiRNA human NBS1 or human CBX5 (Sigma-Aldrich) oligo and the Lipofectamine RNAiMAX transfection reagent (Thermo Fisher Scientific, Waltham, MA, USA)^66^, according to the manufacturer’s instructions. Control cells were double transfected using 5 nM of the Mission® esiRNA Universal Negative Controls (Sigma-Aldrich). Cells were analyzed 48 h after the second transfection by immunoblot experiments.

### CHX treatment of human cells

Human MRC5 cells, either transiently silenced with siNBS1 or with the negative controls oligo, were treated with 100 μg/mL CHX (Sigma-Aldrich) for 1, 2, 4, and 8 h. Protein extracts were obtained from each time point and analyzed by WB.

### MG132 and chloroquine treatments of Drosophila larval brains

Treatment of third instar larval brains either with MG132 or chloroquine was performed by dissecting brains in physiological solution (0.7% NaCl). Brains were then incubated for 4–5 h at room temperature (RT) in 0.5 mL of serum-free Schneider’s insect medium with 200 μM MG132 (Calbiochem, San Diego, CA, USA) or 300 μM chloroquine (Sigma-Aldrich). Afterwards, brains were collected, flash-frozen with liquid nitrogen, and stored at −80 °C until they were analyzed by WB.

### Dephosphorylation of human cell lysates with AP

Five hundred micrograms of whole-protein lysates were incubated for 1 h at 37 °C with 10 U of AP (Thermo Fisher Scientific). The reaction was stopped by the addition of 10 mM orthovanadate. As a control, 1 mg of protein lysate was incubated in the same conditions, in the absence of AP and in the presence of orthovanadate. The untreated and dephosphorylated samples were then immunoprecipitated as described below.

### Antibodies generation against Drosophila Nbs and Mre11

To obtain the anti-Nbs antibody, mice were immunized with a 6 × His-tagged C-terminal polypeptide of Nbs encompassing amino acids 500–818 (UniProtKB/TrEMBL: Q9VT40_DROME). The anti-Mre11 antibody was generated by immunization of mice with a 6 × His-tagged Mre11 C-terminal polypeptide encompassing amino acids 283–620 (UniProtKB/TrEMBL: Q9XYZ4_DROME). The resulting antisera were affinity-purified by standard methods. Mouse immunization and antisera affinity purification were carried out by Agro-Bio (La Ferté St Aubin, France).

### Chromosome cytology, immunostaining, and microscopy

DAPI (4,6-diamidino-2-phenylindole)-stained, colchicine-treated Drosophila larval brain chromosome preparations for the analysis of chromosome aberration were made as previously described^65,67^. Anti-HP1a immunostaining on Drosophila neuroblasts was carried out as described in ref. ^68^. The anti-HP1a antibody C1A9 (dilution 1:300) was obtained from the Developmental Studies Hybridoma Bank, created by the NICHD of the NIH and maintained at the University of Iowa (Department of Biology, Iowa City, IA 52242). The anti-Nbs antibody (this study) was used at a 1:10 dilution. Fluorescein isothiocyanate-conjugated goat anti-mouse (1:20; Jackson Laboratories, Bar Harbor, ME, USA) was used as secondary antibody. All slides were then mounted in VECTASHIELD® Antifade Mounting Medium with DAPI (Vector, Peterborough, UK) to stain DNA. Mitotic chromosome preparations were analyzed using a Zeiss Axioplan epifluorescence microscope (Carl Zeiss, Obezkochen, Germany) equipped with a cooled CCD camera (Photometrics, Woburn, MA, USA).

For immunostaining in human cells, cells were fixed in 4% paraformaldehyde for 10 min on ice, permeabilized with 0.2% Triton X-100/PBS, and then blocked in 3% bovine serum albumin (BSA)/PBS for 1 h at RT. Slides were incubated overnight (ON) at 4 °C with anti-HP1α (1:100; GA-62; Santa Cruz, Dallas, TX, USA), anti-γ-H2AX (1:200; JBW301; Millipore, Billerica, MA, USA), and anti-53BP1 (1:100; MAB3802; Millipore) antibodies diluted 1:100 in 1% BSA/PBS. Slides were then washed and incubated for 1 h at 37 °C with Alexa Fluor 488-conjugated goat anti-mouse IgG or Alexa Fluor 610-conjugated goat anti-rabbit (Immunological Sciences, Rome, Italy) diluted 1:100 in 1% BSA/PBS. Confocal analysis was performed using the LCS Leica confocal microscope (Leica Microsystems, Heidelberg, Germany). HP1α fluorescence intensity was measured by the ImageJ software. The quantitative analysis of γ-H2AX foci/cell was carried out by counting foci in 100 cells/experimental point in three independent experiments.

### RNA extraction, cDNA amplification, and semi-qPCR

Total Drosophila RNA was isolated from third instar larval brains (25 brains/sample) using TRIzol (TRI Reagent^®^ SIGMA Life Science, Sigma-Aldrich) and genomic DNA was eliminated with Invitrogen^TM^ Dnase I Amplification Grade (Thermo Fisher Scientific) by following the manufacturer’s manual. To quantify the expression levels of *Su(var)205* transcripts, equal amounts of cDNA were synthesized from 300 ng of total RNA for each sample by using the iScript™ cDNA Synthesis Kit (Bio-Rad, Hercules, CA, USA). Thirty nanograms of cDNA per reaction were analyzed for semi-qPCR using the SsoAdvanced™ Universal SYBR® Green Supermix Kit (Bio-Rad) following the manufacturer’s protocol. The thermal cycling conditions were: 50 °C (2 min), 95 °C (10 min) followed by 40 cycles at 95 °C (15 s), 60 °C (1 min), and 95 °C (15 s), 60 °C (1 min) 95 °C (15 s), and 60 °C (15 s). The specificity of the reaction was verified by melting curve analysis. The PCR primers used for *Su(var)205* transcripts were: HP1a_FW and HP1a_RV (Supplementary Table 1). The RP49 reference transcript was amplified using primers RP49_FW and Rp49_RV (Supplementary Table 1). Experiments were performed using an ABI Prism 7300 System (Applied Biosystems, Foster City, CA, USA), and data processing was performed using the ABI SDS v2.1 software (Applied Biosystems).

Total human RNA was isolated from cultured cells using TRIzol^®^ Reagent (Ambion Life Technologies, Carlsbad, CA, USA). qPCRs in human cells were performed by reverse transcribing 1 μg of RNA using an oligo-dT primer and the SuperScript® II Reverse Transcriptase system (Life Technologies). qPCR was performed using the iTaq Universal SYBR^®^ Green Supermix (Bio-Rad) as detection system. The thermal cycling conditions were: 95 °C for 2 min followed by 30 cycles at 95 °C for 10 s and 60 °C for 30 s. The PCR primers used were: HP1α_FW, HP1α_RV, β-actin_FW, and β-ACTIN_RV (Supplementary Table 1). Experiments were performed using the 7900HT Fast Real-Time PCR instrument (Applied Biosystems).

The threshold crossing value was noted for each transcript and normalized to the internal control. The fold change was calculated using the comparative 2^(−ΔΔCt)^ method.

### GST-pulldown

To obtain the constructs for the bacterial expression of GST-tagged full-length (FL) HP1a (1–206) and truncated forms of HP1a, namely, GST-CSD (136–206) and GST-ΔCSD (1–141), HP1a-encoding cDNAs were PCR-amplified by using specific primers (see below), which allowed the insertion of 5’ and 3’ 15 bp plasmid complementary sites required for cloning in pGEX6P1 vector (GE healthcare, Chicago, IL, USA) by using the In-Fusion HD Cloning Kit (Takara Bio Inc., Kusatsu, Shiga, Japan). In-Fusion reactions were performed as suggested by the producer’s manual and were then transformed into *Escherichia coli* competent cells. The I191E and W200A point mutations in HP1α were introduced with the QuikChange II XL Site directed Mutagenesis Kit (Stratagene, San Diego, CA, USA) according to the manufacturer’s protocol by using pGEX6P1-HP1a, pGEX6P1-CSD, pENTR-HP1a, pAWH-HP1a, and pAWF-HP1a constructs as a template. The sequence of all resulting recombinant plasmids was finally verified by standard sequencing. The primers used were referred to as HP1a_GST_FW, HP1a_GST _RV, CSD_GST _FW, CSD_GST _RV, ΔCSD_GST _FW, ΔCSD_GST _RV, I191_GST _FW, I191_GST _RV, W200_GST _FW, and W200_GST _RV (Supplementary Table 1).

GST-fusion proteins were obtained by transforming *E. Coli* Rosetta pLys DE3 strain with cDNAs of wild-type, mutant, and truncated forms of HP1 cloned in pGEX6p1 plasmid. The expression of recombinant proteins was induced with IPTG 0.5 mM, 3.5 h at 37 °C at 225 rpm. Bacterial cells were pelleted and resuspended in GST lysis buffer (50 mM Tris-HCl pH 7.4, 100 mM NaCl, 1 mM EDTA, 10% glycerol, 1 mM dithiothreitol (DTT), and protease inhibitor cocktail). After sonication, the lysates were centrifuged at 13,000 rpm 30 min at 4 °C and clarified lysate was next incubated with glutathione Sepharose beads (GE) and purified as described in the manual. The beads were extensively washed three times with PBT (SIGMA Dulbecco’s phosphate buffered saline (PBS 1×) with 1% triton), two times with PBS 1×, resuspended 1:5 in the storage buffer (PBS 1× with 62.5% glycerol), and stored at −20 °C. For GST-pulldown assays, GST fusion proteins, at the final concentration of 4 μM, were preincubated 1 h at 4 °C with 1% BSA in PBT, washed two times with PBT, and incubated with 30 μg of S2 cell extracts in IP buffer (50 mM Tris-HCl pH 7.4, 150 mM NaCl, 1% Triton, 1 mM EDTA, and PIC50X EDTA-free (Roche)) with 10% glycerol, 2 h at 4 °C in agitation. After the incubation, GST proteins were harvested, washed four times with PBT, resuspended in 2× Laemmli Buffer, and analyzed by WB.

### Protein extracts, WB, and antibodies

Protein extracts from Drosophila larval brains were obtained by dissecting larval brains in 0.7% NaCl, and homogenizing them on 2× Laemmli buffer. S2 cells extracts for GST-pulldown analyses and co-IP experiments were obtained by lysing cells for 30 min on ice in IP buffer (50 mM Tris-HCl pH 7.5, 150 mM NaCl, 1%, 1 mM EDTA, Triton, and EDTA-free protease inhibitor cocktail (Roche, Basilea, Switzerland). After sonication (10 s, 10% amplitude), lysates were cleared by 20 min centrifugation at 13,000 rpm at 4 °C and stored after addition of 10% glycerol.

Protein samples were run into sodium dodecyl sulfate-polyacrylamide gel electrophoresis (SDS-PAGE) and blotted on a nitrocellulose membrane (Hybond ECL, Amersham). Filters were blocked in 5% non-fat dry milk dissolved in 0.1% Tween-20/PBS for 30 min at RT and then incubated with the following primary antibodies: mouse HP1a (1:7000; C1A9-2ea), α-HA horseradish peroxidase (HRP)-conjugated (1:5000, GE Healthcare), α-ACT HRP-conjugated (1:10,000; GE Healthcare), anti-Rad50 (1:1000; ref. ^16^), anti-Nbs and anti-Mre11 (1:500; this study), and anti-Giotto (1:5000; ref. ^69^) ON at 4 °C. After three washes with 0.1%Tween-20/PBS, membranes were incubated with anti-rabbit or anti-mouse HRP-conjugated secondary antibodies (1:5000; GE Healthcare) for 1 h at RT and then washed again 3 times with 0.1% Triton X containing PBS.

Protein extracts from human cell lines were obtained by lysing cells in 8 M urea, 15 mM β-mercaptoethanol, 50 mM Tris-HCl pH 7.5, 1 mM DTT, and protease inhibitors. Protein extracts were quantified using Bradford protein assay (Bio-Rad), resolved by SDS-PAGE, and transferred to polyvinylidene fluoride membranes. Membranes were blocked for 40 min at RT with 5% nonfat dry milk/0.1% Tween-20/PBS, probed first with primary antibodies for either 2 h at RT or ON at 4 °C, and finally with the appropriate HRP-conjugated secondary antibody for 1 h at RT. The primer antibodies and the corresponding dilutions used in WBs were: anti-HP1α (1:1000; GA-62), anti-NBS1 (1:1000; A-2), anti-NBS1 (1:1000; B-5), anti-vinculin (1:10000; 7F9), and anti-β actin (1:10000; C-2) from Santa Cruz Biotechnology; anti-RAD50 (1:1000; 3427; Cell Signaling technology, Danvers, MA, USA).

The chemiluminescent signal was revealed through either ECL (Thermo Fisher Scientific) or Clarity^TM^ Western ECL substrate (Bio-Rad) using the ChemiDoc scanning system (Bio-Rad). Band intensities were quantified using the Image Lab 4.0.1 software (Bio-Rad). WBs were repeated at least three times.

### Immunoprecipitation

For IP of S2 cell line extracts, transiently transfected S2 cells with the HP1-FLAG expression vector were lysed as described above in 300–600 μL IP buffer and immunoprecipitated with the proper affinity matrix (anti-FLAG M2 affinity agarose gel, Sigma). Affinity matrix was preincubated 1 h at 4 °C with 1% BSA in PBT and washed 3 times with PBT. S2 cell extracts were incubated in agitation with the matrix 2 h at 4 °C. Matrix-bound proteins were washed four times with PBT, boiled in 2× Laemmli buffer and analyzed by immunoblotting.

For IP of human cell line extracts, whole-cell lysates (concentration 5 μg/μL) were first precleared using 20 μL of protein A/G PLUS-agarose beads (Santa Cruz) for 1 h at 4 °C and then incubated with either 0.1 μg/μL anti-HP1α or 0.2 μg/μL anti-NBS1 (A-2, raised against amino acids 1–300 mapping at the N-terminus of NBS1; Santa Cruz) or 0.2 μg/μL anti-NBS1 (B-5, specific for an epitope mapping between amino acids 731 and 754 at the C-terminus of NBS1; Santa Cruz) for 4 h at 4 °C. Finally, 20 μL of protein A/G PLUS-agarose beads were added to the mixture and incubated ON at 4 °C. Immunoprecipitates were washed with the NP-40 lysis buffer (20 mM Tris-HCl pH 8.0, 137 mM NaCl, 10% glycerol, 1% NP-40, 10 mM EDTA) and resolved on an SDS-PAGE. Experiments were repeated at least three times.

### Statistical analysis

Data were expressed as mean values ± standard deviation (SD) of at least three experiments. Statistical analysis was performed using Student’s *t* test. The results were considered significant when *p* values were <0.05.

### Bioinformatics analysis

The three-dimensional model of the Nbs1 peptide spanning the 332–347 region of the protein has been built with Swiss-PDB viewer^70^ using as a template the structure of the Shugoshin 1 peptide bound to the HP1α CSD^28^. Molecular docking simulations between HP1α (PDB code: 3Q6S) and Nbs1 peptide phosphorylated and non-phosphorylated on Ser343 have been performed using the protein–protein docking program ZDOCK^71^ (https://zdock.umassmed.edu; version 3.0.2), which employs a Fast Fourier Transform algorithm and a scoring system based on a combination of shape complementarity, electrostatics, and statistical potential terms. The 2000 complexes generated by ZDOCK were re-ranked using ZRANK^72^, which uses a more detailed potential including electrostatics, van der Waals, and desolvation terms. Electrostatic potential calculations have been carried out using the DelPhi software for solving the Poisson–Boltzmann equation^73^. The models of the complexes formed by HP1α with the Nbs1 332–347 region have been analyzed and the corresponding images were generated using the UCSF Chimera package^74^.

## Supplementary information

SUPPLEMENTARY MATERIAL

SUPPLEMENTARY FIGURE 1

SUPPLEMENTARY FIGURE 2

SUPPLEMENTARY FIGURE 3

SUPPLEMENTARY FIGURE 4

SUPPLEMENTARY FIGURE 5

SUPPLEMENTARY FIGURE 6

SUPPLEMENTARY FIGURE 7

SUPPLEMENTARY FIGURE 8

SUPPLEMENTARY FIGURE 9

SUPPLEMENTARY TABLE 1
