# Supplementary material for: NBS1 interacts with HP1 to ensure genome integrity
Source: Cell Death Dis. 2019 Dec 13;10(12):951. doi: 10.1038/s41419-019-2185-x (PMC6911104; doi:10.1038/s41419-019-2185-x)
Supplement: Supplementary file 1 — SUPPLEMENTARY MATERIAL [file 41419_2019_2185_MOESM1_ESM.docx]

**NBS1 interacts with HP1 to ensure genome integrity**

Giuseppe Bosso^1,3,4,8^, Francesca Cipressa^1,3,8^, Maria Lina Moroni^1^, Rosa Pennisi^2^, Jacopo Albanesi^2^, Valentina Brandi^2^, Simona Cugusi^5^, Fioranna Renda^6^, Laura Ciapponi^1^, Fabio Polticelli^2,7^, Antonio Antoccia^2,7^, Alessandra di Masi^2,*^ , Giovanni Cenci^1,3,*^

**Supplementary Figure and Table Legends**

**Supplementary Figure 1.** **Loss of HP1a does not affect MRN protein levels in *Drosophila*.** WB from either control (-) or *Su(var)2-5 RNAi* interfered (+) larval brain extracts and hybridized with anti-HP1a, anti-Mre11, anti-Rad50, and anti-Nbs1 antibodies. Anti-Giotto has been used as a loading control. Asterisks indicate aspecific bands

**Supplementary Figure 2**. **Reduction of HP1a levels in *nbs* mutants is rescued by lysosome inhibition.** WB from control (OR) and *nbs* mutant extracts revealed with the anti-HP1a antibody following MG132 and chloroquine treatment. Anti-Giotto has been used as a loading control.

**Supplementary Figure 3. Chromosome breaks in *nbs* mutant chromosomes**. DAPI-stained colchicine-treated neuroblast metaphases from wild-type (OR)(a) and either *nbs^1^* (b) or *nbs^1^, Su(var)205-RFP* (c) mutants. Note that depletion of Nbs induces the formation of frequent chromosome breaks (b) that are reduced upon co-expression of HP1a-encoding *Su(var)2-5* gene (c). Numbers indicate the different chromosomes of a normal Drosophila karyotype. Arrowheads indicate a double strand break involving both chromatids of chromosome 3 (chromosome break). Arrows show telomere fusions whose frequency is not affected upon expression of HP1a (see text for further information).

**Supplementary Figure 4. Expression of *Su(var)2-5* transcripts in the *MRN* mutants.** qPCR on *Su(var)2-5* mRNAs extracted from *nbs*, *mre11*, and *rad50* mutant brains. Bars indicate ±S.D.

**Supplementary Figure 5. Phosphorylation-dependent interaction between HP1 and NBS1.** One milligram of whole protein lysate obtained from MRC5 cells were incubated for 1 h at 37 °C, in the absence (−) or in the presence (+) of 10 U alkaline phosphatase (AP). The reaction was stopped by the addition of 10 mM orthovanadate. The untreated and dephosphorylated samples were then immunoprecipitated with an anti-HP1 antibody. Ten micrograms of total protein lysate (1%) were loaded as input. Membranes were blotted with anti-NBS1; total IgG levels and vinculin were used as loading control for immunoprecipitates and input, respectively.

**Supplementary Figure 6. *HP1α* mRNA levels in cells with impaired NBS1.** a) qPCR on mRNAs extracted from mocked and NBS1-silenced MRC5 cells, either untreated or irradiated with 1 Gy of X-rays and harvested after 0.5 h. Graphs represent the fold induction of HP1α in irradiated versus normalized untreated cells ± S.D. (*t*-Student’s Test; *p<0.01). b) qPCR on mRNAs extracted from MRC5 and NBS cells. Graphs represent the fold induction of HP1α in NBS cells normalized to MRC5 ± S.D.

**Supplementary Figure 7**. MRC5 cells, either mocked or silenced for NBS1, were treated with 50 μg/mL cycloheximide (CHX) and harvested after, 1, 2, 4, and 8 hours. Membranes were probed with anti-HP1α and anti-NBS1; α-tubulin was used as loading control. Graphs indicate the mean values derived from two independent experiments ±S.D. (*t*-Student’s Test; *p<0.05; **p<0.01; ***p<0.001).

**Supplementary Figure 8.** Representative images of γH2AX (Alexa Fluor 488, green) and 53BP1 (Alexa Fluor 610, red) foci and the corresponding bright field in either mock or siHP1α-treated MRC5 and NBS cells. Cells were irradiated with 1 Gy and fixed at the indicated time points. Magnification ×88.

**Supplementary Figure 9**. Ordered and disordered domains of NBS1 have been determined using the FoldIndex software ^91^.

**Supplementary Figure 10. Localization of *Drosophila* Nbs on mitotic chromosomes**. DAPI staining and anti-Nbs immunofluorescence on wild-type (OR-R) and *nbs* mutant chromosomes from third instar larval neuroblast metaphases.

**Supplementary Table 1.** Names and sequences of all primers used in this study.
