# Supplementary figures and images for: NBS1 interacts with HP1 to ensure genome integrity
Source: Cell Death Dis. 2019 Dec 13;10(12):951. doi: 10.1038/s41419-019-2185-x (PMC6911104; doi:10.1038/s41419-019-2185-x)

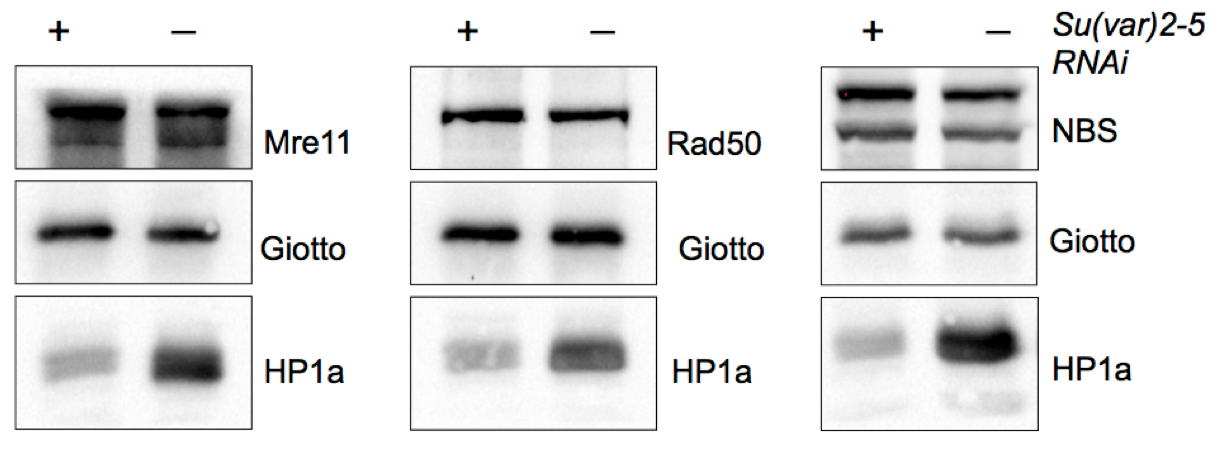

Supplement: Supplementary file 2 — SUPPLEMENTARY FIGURE 1 [file 41419_2019_2185_MOESM2_ESM.png]

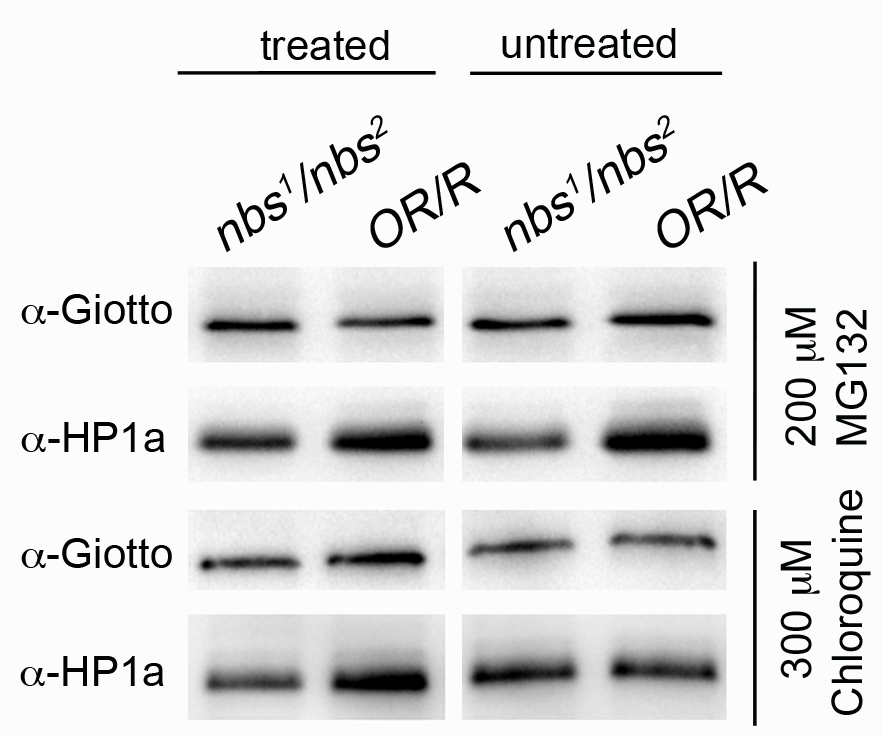

Supplement: Supplementary file 3 — SUPPLEMENTARY FIGURE 2 [file 41419_2019_2185_MOESM3_ESM.png]

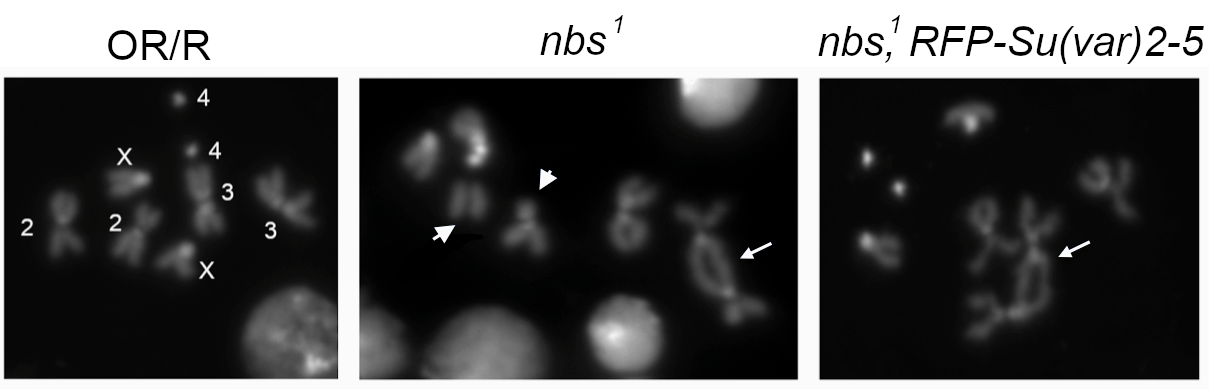

Supplement: Supplementary file 4 — SUPPLEMENTARY FIGURE 3 [file 41419_2019_2185_MOESM4_ESM.png]

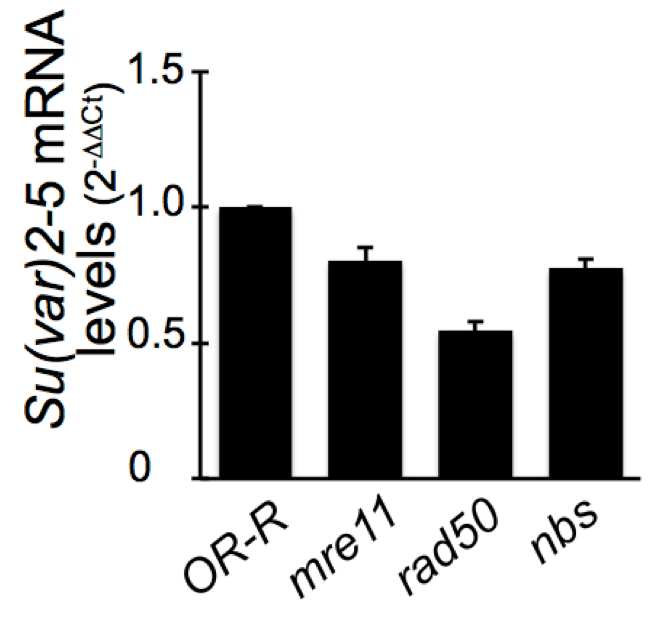

Supplement: Supplementary file 5 — SUPPLEMENTARY FIGURE 4 [file 41419_2019_2185_MOESM5_ESM.png]

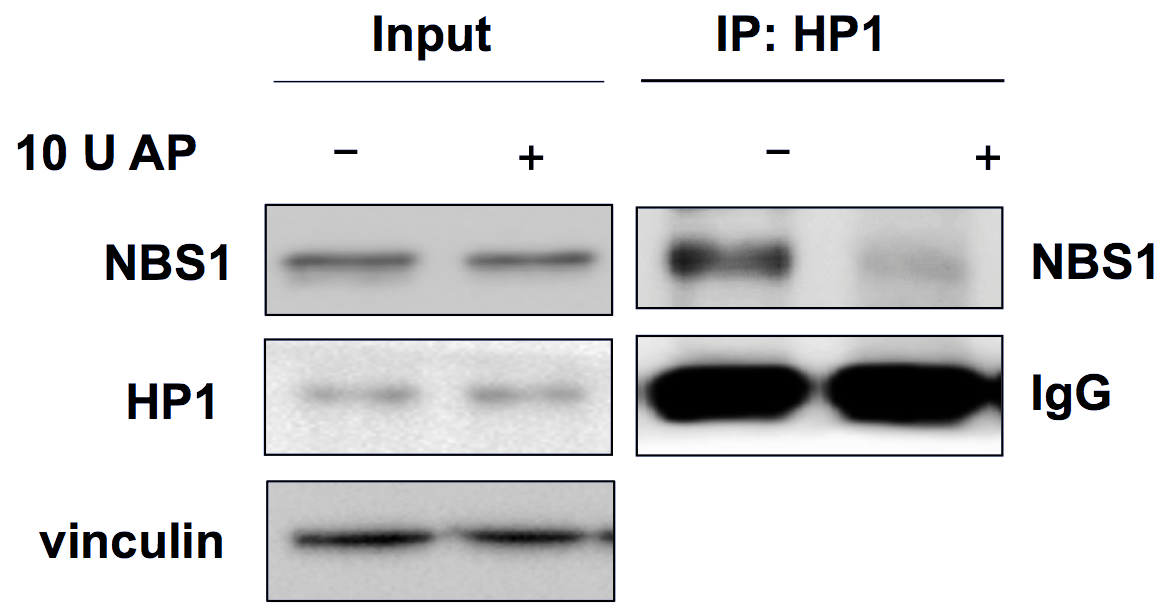

Supplement: Supplementary file 6 — SUPPLEMENTARY FIGURE 5 [file 41419_2019_2185_MOESM6_ESM.png]

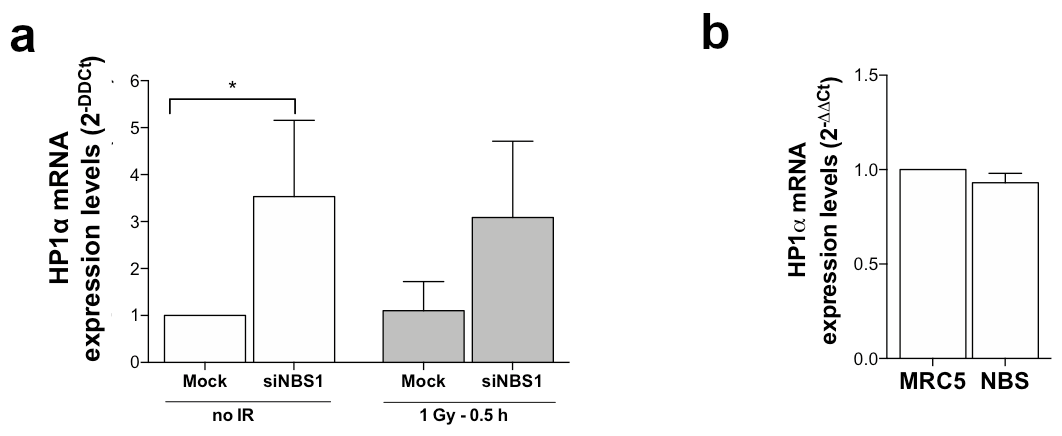

Supplement: Supplementary file 7 — SUPPLEMENTARY FIGURE 6 [file 41419_2019_2185_MOESM7_ESM.png]

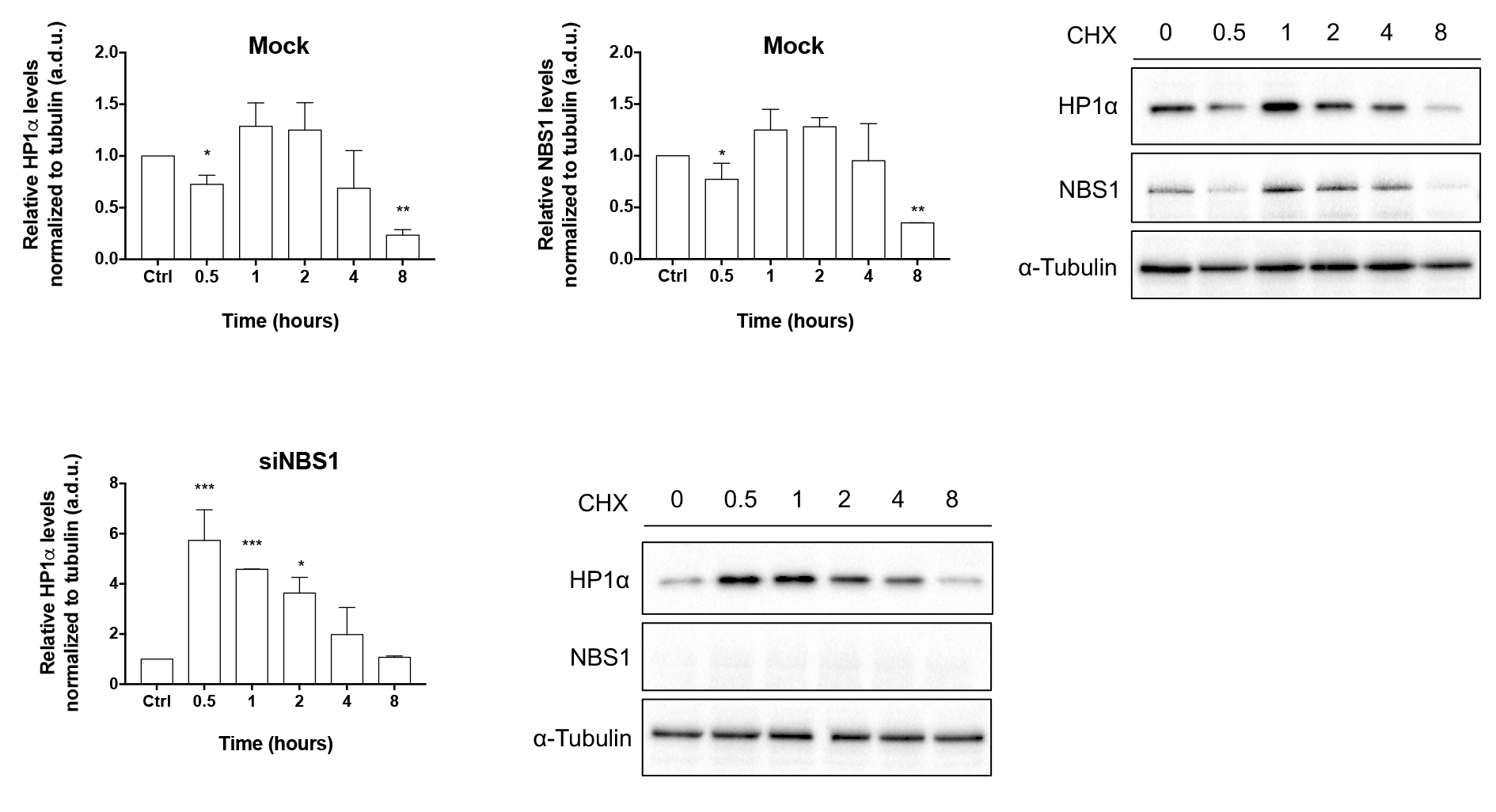

Supplement: Supplementary file 8 — SUPPLEMENTARY FIGURE 7 [file 41419_2019_2185_MOESM8_ESM.png]

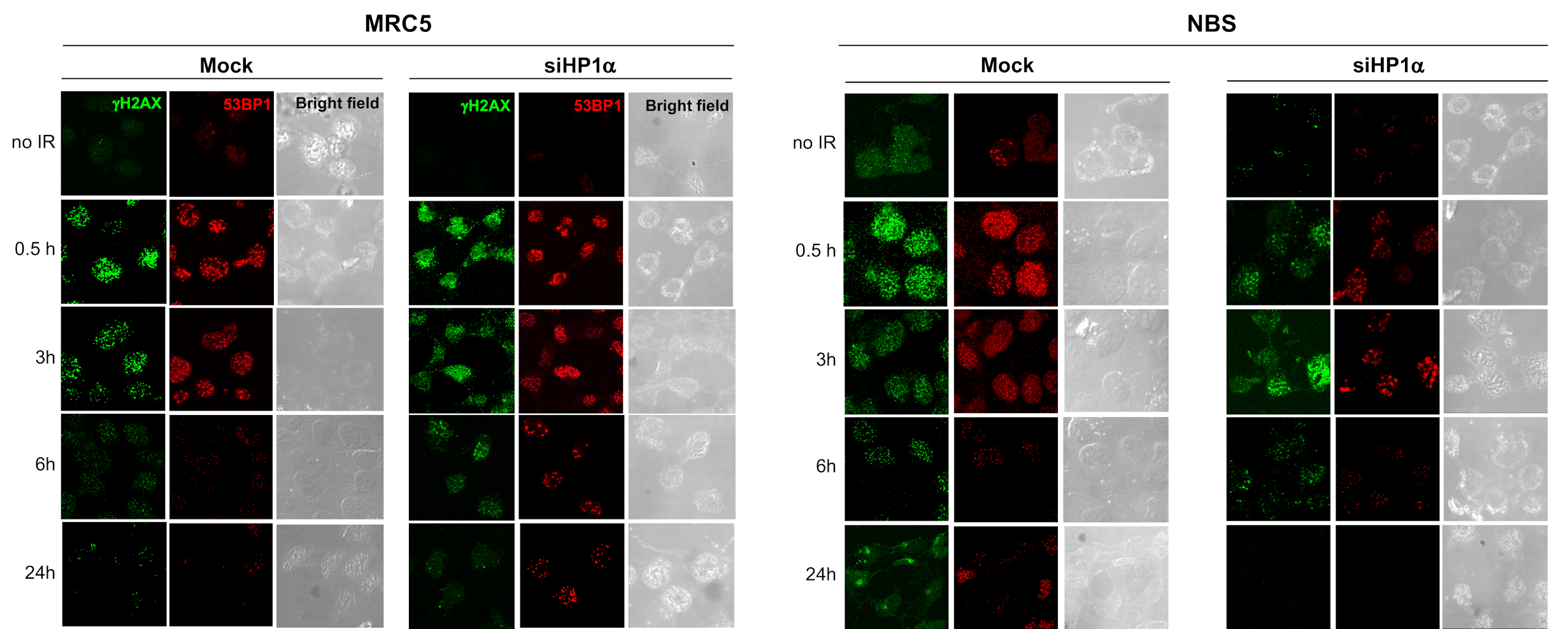

Supplement: Supplementary file 9 — SUPPLEMENTARY FIGURE 8 [file 41419_2019_2185_MOESM9_ESM.png]

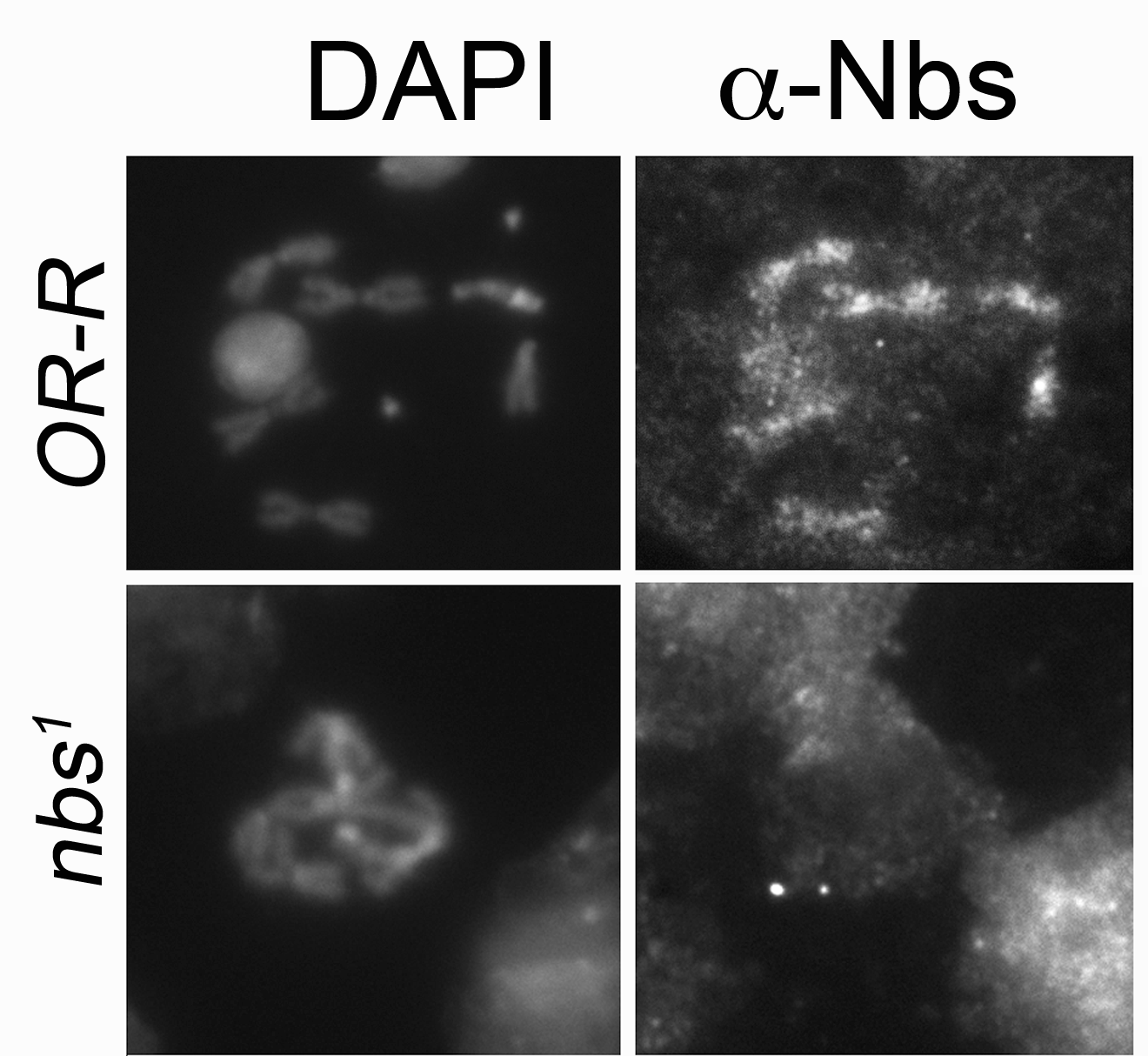

Supplement: Supplementary file 10 — SUPPLEMENTARY FIGURE 9 [file 41419_2019_2185_MOESM10_ESM.png]
