# Supplementary material for: NBS1 interacts with HP1 to ensure genome integrity
Source: Cell Death Dis. 2019 Dec 13;10(12):951. doi: 10.1038/s41419-019-2185-x (PMC6911104; doi:10.1038/s41419-019-2185-x)
Supplement: Supplementary file 11 — SUPPLEMENTARY TABLE 1 [file 41419_2019_2185_MOESM11_ESM.docx]

| **Name** | **Sequence** |
| --- | --- |
| *nbs_FW:* | 5’-ATGTTTGTTTTGACTAAAGATGAC-3’, |
| *nbs_RV* | 5’-AACACAGGTTACCAACCATC-3’ |
| *HP1a_FW:* | 5’-GAGAAGTCAGCCGCCTCCAA-3’ |
| *HP1a_RV:* | 5’-CGGACTTTCGCTTGCTTGCT-3’. |
| *RP49_FW* | 5’-CCGCTTCAAGGGACAGTATCT-3’ |
| *Rp49_RV* | 5’-ATCTCGCCGCAGTAAACGC-3’. |
| *HP1α_FW:* | 5’-CGCTCTGACCTAAACTGCTC-3’, |
| *HP1α_RV* | 5’-ACCTTCTCCACAACATACTCC-3’ |
| *β-ACTIN_FW* | 5’-AGAGGGAAATCGTGCGTGAC-3’ |
| *β-ACTIN_RV* | 5’-CAATGGTGATGACCTGGCCG-3’. |
| *HP1a_GST_FW:* | 5’-GGGATCCCCGGAATTCATGGGCAAGAAAATCGACAACCCTGAG-3’ |
| *HP1a_GST _RV:* | 5’-AGTCACGATGCGGCCGCTTAATCTTCATTATCAGAGTACCAGGATAGGCG-3’, |
| *CSD_GST _FW* | 5’-GGGATCCCCGGAATTCCCCGTTTCAGGATCTACCGGATTC-3’ |
| *CSD_GST _RV:* | 5’-AGTCACGATGCGGCCGCTTAATCTTCATTATCAGAGTACCAGGATAGGCG-3’ |
| *ΔCSD_GST _FW:* | 5’-GGGATCCCCGGAATTCATGGGCAAGAAAATCGACAACCCTGAG-3’ |
| *ΔCSD_GST _RV:* | 5’-AGTCACGATGCGGCCGCTTAGGTAGATCCTGAAACGGGAATGGTGTC-3 |
| *ΔCD_GST _FW:,* | 5’-GGGATCCCCGGAATTCCCCAGCAGCAGCGCCAAGG-3’ |
| *ΔCD_GST _RV:* | 5’-AGTCACGATGCGGCCGCTTAATCTTCATTATCAGAGTACCAGGATAGGCG-3’ |
| *CD_GST _FW:,* | 5’-GGGATCCCCGGAATTCATGGGCAAGAAAATCGACAACCCTGAG-3’ |
| *CD_GST _RV:* | 5’-AGTCACGATGCGGCCGCTTATCGATCCTTCTTGGAGGCGGCTGA-3’ |
| *I191_GST _FW* | 5’-CCCACGAATGGTAGAGCACTTCTACGAAGAGCGCC-3’ |
| *I191_GST _RV:* | 5’-GGCGCTCTTCGTAGAAGTGCTCTACCATTCGTGGG-3’ |
| *W200_GST _FW:* | 5’-CTACGAAGAGCGCCTATCCGCGTACTCTGATAATGAAG-3’ |
| *W200_GST_RV:* | 5’-CTTCATTATCAGAGTACGCGGATAGGCGCTCTTCGTAG-3’ |
